# Data congruence, paedomorphosis and salamanders

**DOI:** 10.1186/1742-9994-4-22

**Published:** 2007-10-31

**Authors:** Torsten H Struck

**Affiliations:** 1 Department of Biology/Chemistry, University of Osnabrück, Barbarastr. 11, Osnabrück, D-49076, Germany

## Abstract

**Background:**

The retention of ancestral juvenile characters by adult stages of descendants is called paedomorphosis. However, this process can mislead phylogenetic analyses based on morphological data, even in combination with molecular data, because the assessment if a character is primary absent or secondary lost is difficult. Thus, the detection of incongruence between morphological and molecular data is necessary to investigate the reliability of simultaneous analyses. Different methods have been proposed to detect data congruence or incongruence. Five of them (PABA, PBS, NDI, LILD, DRI) are used herein to assess incongruence between morphological and molecular data in a case study addressing salamander phylogeny, which comprises several supposedly paedomorphic taxa. Therefore, previously published data sets were compiled herein. Furthermore, two strategies ameliorating effects of paedomorphosis on phylogenetic studies were tested herein using a statistical rigor. Additionally, efficiency of the different methods to assess incongruence was analyzed using this empirical data set. Finally, a test statistic is presented for all these methods except DRI.

**Results:**

The addition of morphological data to molecular data results in both different positions of three of the four paedomorphic taxa and strong incongruence, but treating the morphological data using different strategies ameliorating the negative impact of paedomorphosis revokes these changes and minimizes the conflict. Of these strategies the strategy to just exclude paedomorphic character traits seem to be most beneficial. Of the three molecular partitions analyzed herein the RAG1 partition seems to be the most suitable to resolve deep salamander phylogeny. The rRNA and mtDNA partition are either too conserved or too variable, respectively. Of the different methods to detect incongruence, the NDI and PABA approaches are more conservative in the indication of incongruence than LILD and PBS.

**Conclusion:**

Paedomorphosis induces strong conflicts and can mislead the phylogenetic analyses even in combined analyses. However, different strategies are efficiently minimizing these problems. Though the exploration of different methods to detect incongruence is preferable NDI and PABA are more conservative than the others and NDI is computational less extensive than PABA.

## Background

The amount of molecular and morphological data used in phylogenetic reconstructions is steadily increasing in the past decades [e.g., [1,2]]. Thus, phylogenies based on multiple data sets (i.e., partitions) bearing on both molecular and morphological data are common nowadays [e.g., [2-4]]. However, conclusions regarding phylogenetic relationships based on either morphological or molecular data do not always result in congruent phylogenies [e.g., [2,5-12]]. Molecular evolutionary events like genome fusion, gene duplication, horizontal gene transfer, heterotachy or gene extinction or saturation and model misspecifications disconnect gene trees in part from species trees [e.g., [13-18]]. On the other hand, in morphological data sets signal-like pattern due to other processes than speciation can also occur and thus disconnect species trees from morphological trees. The assessment of absent characters is especially difficult for morphological characters: Have they never evolved in these species or are they secondary lost [e.g., [19-21]]? The problem of primary absence or secondary loss is well known for taxa of supposedly paedomorphic origin [2,22]. The retention of ancestral juvenile characters by adult stages of descendants is called paedomorphosis [23,24]. Paedomorphosis can arise either by a retardation of somatic development (neoteny) or by an acceleration of sexual maturation (progenesis) [23]. Thus, such adults lack several characters exhibited in adults of non-paedomorphic sister taxa. Paedomorphic evolution has been assumed for several taxa from different metazoan clades such as polychaetes or tetrapods [e.g., [2,6,7,22,25-36]]. However, usually always the reconstruction of their phylogenetic position is hampered using morphological data due to the absence of comparable adult character traits [see [2,22]]. Molecular data nearly always result in different phylogenetic positions [see [2,22]]. Comparing incongruent branches from molecular and morphological data sets and examining the evolution of individual morphological characters Wiens et al. [2] showed the problems in morphological cladistic analyses of paedomorphic taxa. However, the strength of the conflict induced by paedomorphosis was not assessed by any statistical or methodological means. In their combined analyses strategies were adopted to circumvent the problems introduced by paedomorphosis. Wiens et al. [2] coded the adult morphology of paedomorphic taxa as unknown, because adult, sexually mature stages of paedomorphic and non-paedomorphic species are not comparable ontogenetic stages. Though this strategy might be overly conservative by deleting characters not affected by paedomorphosis Wiens et al. [2] favoured this strategy over another strategy, which was also adopted by them as well as other authors [e.g., [33]]. This second strategy excludes supposed paedomorphic character traits. However, this means that paedomorphic character traits must be identified *a priori*, which may be difficult. Furthermore, Wiens et al. [2] showed that other character traits such as bone structures can also have a negative influence on reconstructions of phylogenetic trees due to convergent adaptation to the same aquatic habitat or the lack of metamorphosis and thus of 'adult' apomorphies. However, priority of the first strategy over the second one was not tested using a statistical rigor.

Salamanders provide a good and actually the only model system to date to analyze the impact of paedomorphosis on phylogenetic reconstructions. Only a few other metazoan taxa have similar numbers of paedomorphic taxa. However, none of them is adequate for such a study in the moment. For example, the dorvilleid annelids have several paedomorphic genera, which are most times very small in size with less than one mm [9,22]. Eibye-Jacobsen & Kristensen [9] conducted thorough morphological cladistic analyses covering all dorvilleid genera. However, their data set contained only 38 characters in contrast to the 326 morphological characters of Wiens et al. [2]. Furthermore, it cannot be expected that this number can be increased much more, because the matrix of the dorvilleids is already comprehensive regarding the number of possible characters. Though first preliminary molecular analyses based on rRNA genes indicate that strong incongruence between morphological and molecular data can also be shown for paedomorphic dorvilleids [5-7,22] no comprehensive molecular data set covering different genes exist to date. Furthermore, the establishment of such a data set cannot be expected in the near future due to both the small size of some taxa and difficulties to collect some of the crucial genera. Besides the comprehensive molecular data set of Wiens et al. [2] consisting of rRNA data published by Larson and Dimmick [35] and their RAG1 sequences Weisrock et al. [37] presented a comprehensive mtDNA data set in a recent study addressing the utility of mitochondrial sequences for salamander phylogeny. This analysis also comprised the rRNA data published by Larson and Dimmick [35], but not the RAG1 data. Thus, both the most comprehensive morphological and molecular data sets for metazoan taxa with paedomorphic species have been collected for salamanders. Interestingly, all molecular analyses of Weisrock et al. [37], including the individual rRNA analyses, recovered phylogenies generally similar to each other, but quite different from the molecular tree presented by Wiens et al. [2], who did not conduct individual analyses of their two molecular partitions. Although Weisrock et al. [37] found that mtDNA might not be able to resolve nodes deep in salamander phylogeny I included their mtDNA nonetheless to further investigate the conflict between the molecular data sets as well as their contributions to combined analyses. For example, can the combined molecular data overwhelm the phylogenetic signal-like pattern in the morphological data set due to paedomorphosis?

### How to detect incongruence?

Thus, the development of tools to detect congruence or incongruence (also called conflict) between partitions is important to test the reliability of reconstructions using concatenated data sets in simultaneous analyses [e.g., [38-40]] and to guide the design of future studies. Albeit given that the term phylogeny can have a far more general connotation, herein it is used for trees obtained from a data matrix using a specific reconstruction method. Furthermore, I also acknowledge that conflict cannot only appear between data sets and their corresponding trees, but also to the fossil record, biogeography, scenarios of organ evolution, model specifications and so on. However, herein conflict is restricted to incongruence between partitions.

Perhaps the most popular method for assessing incongruence in signal-like patterns across partitions is the Incongruence Length Difference (= ILD) test [[41], but see also [42]]. Another recent approach testing for congruence employs reciprocal Shimodaira and Hasegawa [[43], SH] tests comparing trees obtained by different partitions or combinations of partitions [e.g., [42,44,45]]. Both approaches are global in that the overall topology is tested. The taxa or nodes causing incongruence are not revealed. Thus, the tests may be prone to reject congruence between partitions even though the conflicting signal is restricted to a limited number of nodes or taxa [e.g., [44]]. Therefore, employing taxon jackknifing procedures has been suggested as a refinement for both tests [42,44]. This successive taxon deletion approach may be useful for detecting conflict due to a particular terminal node, but it is not guaranteed to find conflict at internal nodes and incongruence resulting from more than one or two terminal nodes [5].

The Relative Apparent Synapomorphy Analysis (RASA) [46] has also been used to determine combinability and phylogenetic signal of data matrices [47-49]. However, Simmons et al. [50] showed that RASA is not able to detect phylogenetic signal and thus in consequence also not congruence or incongruence. Furthermore, RASA does not specify if certain sets of taxa cause the conflict or if the conflict is more evenly distributed. Although usually regarded as an alternative to simultaneous analyses of concatenated partitions super tree or consensus tree methods can be used to investigate congruence between different partitions based on the trees obtained from analyses of the individual partitions [e.g., [51]]. To further visualize the conflict at nodes phylogenetic networks instead of trees can be employed [e.g., [52]]. However, trees obtained only from single partition analyses might not reveal the hidden support or conflict at some nodes, which becomes apparent in simultaneous analyses [e.g., [40,53]]. Furthermore, these approaches do not provide specific values, which can be compared between the nodes.

Approaches utilizing a node-by-node procedure accompanied by specific values substantially increase insights into sources of congruence and conflict. Herein I used approaches based on two different nodal support measurements. The general rationale of these tests is that signal from additional data will increase nodal support for a given node if the evolutionary history of the node is congruent in the partitions. In contrast, incongruent evolutionary histories between partitions at a given node will result in a decrease. Several authors investigated alterations of bootstrap (BP) support to identify the source of congruent or incongruent signal [39,54-59]. However, these investigations were not systematically conducted and only nodes or partitions already suspected of conflict or of *a priori *interest are considered. Reed and Sperling [60] analyzed the effect of two partitions on the butterfly genus *Papilio *phylogeny using differential weights for the partitions. The BP support at each node was investigated for trends as the weight of the EF1α partitions increased relative to the COI/COII partition. However, such analyses are more or less restricted to bipartitioned data sets as weighting schemes become increasingly complicated as numbers of partitions increase and are less straightforward to be analyzed. O'Grady et al. [61] modified this procedure by just downweighting a particular partition to zero. Though proposed for a bipartitioned data set this modification is easily adaptable for data sets with more than two partitions. However, in data sets with only two partitions the procedure of Reed and Sperling [60] is preferable over this modification and the Partition Addition Bootstrap Alteration (PABA) approach of Struck et al. [5], because the latter two are less likely to reveal trends in BP support if only two partitions are defined. Struck et al. [5] developed the PABA approach, which can expose congruence or incongruence, respectively, by examining methodologically the alteration (*δ*) of BP values at a given node when additional data partitions are added. *δ *is examined under all possible combinations of partition addition (both number of partitions and order of addition) to elucidate how all partitions interact with each other:

*PABA*_*n *_= *δ*_*n *_= *BP*_*A *_- *BP*_*B*_

with *n *being the order of addition (e.g., as 2^nd^, 3^rd^, 4^th^), *BP*_*A *_is the BP support after addition of the partition and *BP*_*B *_before addition. For example, partition C is added as 2^nd ^in turn to each other partition in the data set and the alteration of BP support is determined. Then partition C is added as 3^rd ^to all possible combinations of all other partitions comprising only two partitions and so on, till it is added as the last partition to the combined data set of all other partitions. This is repeated for each partition in the data set and each node of interest, usually all nodes of the best tree of the combined data set, but any node can be analyzed. To condense the results the mean values of *δ *are calculated for each partition and order of addition. Thus, the PABA approach is a thorough investigation on a node-by-node and partition-by-partition basis of BP alteration. The principle of analyzing trends of nodal support alteration due to addition of partitions does not depend upon BP values, but can be used for any nodal support measurement. For example, Bond and Hedin [62] implemented a similar procedure using posterior probabilities. For several nodes of the combined data set they determined in which combinations of partitions these nodes were also present and if the posterior probabilities were above 0.90. However, they did assess the actual alteration in posterior probabilities in contrast to the PABA approach. Thus, they utilized a qualitative instead of a quantitative approach. Struck et al. [5] did not provide a procedure to determine how significant the alteration of BP values actually is. Herein, I propose such a procedure based on the generation of random partitions of the same size (see below) [41,63].

Other approaches to investigate congruence or incongruence on a node-by-node basis use another measurement of clade support. Bremer support (BS) values [64-66] were proposed as measurements of support for nodes reconstructed in the most parsimonious tree(s) and thus are equal to or larger than zero. For each node of this tree, the difference in tree length (TL) between the most parsimonious tree and the most parsimonious alternative tree not containing this particular node is determined. Additionally, the definition and use of BS has been extended beyond the nodes obtained in the most parsimonious tree [40,63,67] and thus the values can also be negative. BS values can be calculated using the following general formula:

*BS *= *TL*_*alternative *_- *TL*_*node*_

with *TL*_*node *_being the tree length of the best tree containing the node of interest and *TL*_*alternative *_being the best tree not containing this node. *TL*_*node *_can be determined running analyses constraining the particular node and *TL*_*alternative *_keeping only trees not agreeing with this constraint [e.g., [40,63-65,67]].

Based on the Bremer support [64-66] Baker and DeSalle [68] developed the Partitioned Bremer Support (PBS), which assesses the contribution of each partition to the BS value of the combined analysis of all partitions [40]. Positive PBS values show that the partition is in agreement with the node of interest, but negative values point to a source of conflict. PBS values are calculated in a similar manner as the BS values. As for the BS values the best tree and the best alternative for each node are obtained using the complete data set. However, each partition is separately optimized on these two trees and the difference in tree length is calculated using Equation 2.

The Localized ILD (LILD) test [63] uses directly the BS values of individual partitions. For each node of the combined analysis, which was not recovered in the analysis of an individual partition, Thornton and DeSalle [63] determined the BS value of that partition for that node. Therefore, they obtained only negative BS values. Herein, I used an extended definition of the LILD test to include all nodes regardless whether they were obtained in the analysis of an individual partition or not. In essence, these are all the BS values of an individual partition for the set of nodes analyzed and thus they can be positive, zero or negative.

In contrast to PBS and LILD, which are based on the analysis of individual partitions, the Nodal Data set Influence (NDI) approach [40] determines the alteration of BS values at nodes as a partition is removed from the complete data set. Thus, NDI not only assesses the contribution of the individual partition to the BS support of the combined data set, but also the contribution the partition brings out of the other partitions in simultaneous analyses [40]. NDI is in its calculation similar to the highest order of partition addition in PABA. As mentioned above, PABA does not depend on the nature of the nodal support measurement. Therefore, besides NDI I also determined the alteration of BS values at lower orders of partition addition (i.e., as 2^nd ^and 3^rd^). In accordance with PABA this approach is called Partition Addition Bremer Support Alteration (PABSA) and for a total of four partitions PABSA_4 _is synonymous with NDI:

*NDI/PABSA*_*n *_= *BS*_*A *_- *BS*_*B*_

with *n *being the order of addition (e.g., as 2^nd^, 3^rd^, 4^th^), *BS*_*A *_is the BS value after addition of the partition and *BS*_*B *_before addition.

Finally, analogous to the clade stability index [69], which is too time consuming to be computed in larger data sets because it is based on the removal of single characters, Gatesy et al. [40] introduced the Data set Removal Index (DRI). DRI is the minimum number of partitions to be removed to collapse a node obtained in the analysis of all partitions. For nodes not recovered in the combined analysis DRI is always zero. The DRI provides a measurement of how many and which data sets are necessary to recover a node in a simultaneous analysis. The higher the DRI the more partitions agree with the node. DRI has an upper limit with the number of partitions defined.

### Determination of significance

To test the significance of PBS and BS values several authors suggested employing the Templeton [70] test to examine clade significance [71-74]. However, strictly spoken this test does not measure the significance of the actual values, but the differences of the underlying trees. The Templeton [70] test compares two trees to each other by assessing at each position which of the two trees is favored and to what degree. Then a Wilcoxon signed rank test (WRST) is used to determine the significance of the obtained differences in support. It remains unresolved if the compared trees are *a priori *or *a posteriori *hypotheses, in other words, if they were postulated before or after the phylogenetic reconstruction [74]. Results of Templeton [70] tests are only reliable if two *a priori *hypotheses are compared to each other [75]. Finally, the test cannot be adopted for the PABA and NDI/PABSA approaches.

Thornton and DeSalle [63] adopted the procedure of the ILD test to assess significance [41] for their LILD test. The test procedure generates new partitions of same sizes as the defined partitions by randomly assigning positions from the complete data set to partitions. Thus, the procedure can assess if the same value can be obtained just by chance due to randomly partitioning the data set in partitions of same sizes as defined partitions. Except for DRI it can be adopted for all approaches used herein. Therefore, herein significance with respect to values obtained by PBS, NDI/PABSA, LILD or PABA means that this value could not have been obtained by randomly partitioning the data set.

In contrast to the ILD test however, the two-sided tail probability has been determined herein. In all approaches used herein the values can be either positive, zero or negative. Randomly partitioning the data set and determining the specified values (e.g., NDI) will generate a distribution of these values for which it cannot be predict beforehand if our test value will be on the right-hand or left-hand side of the mean value, which is not necessarily zero. Therefore, the two-sided tail probability has been determined. On the other hand, the values of the ILD test can never be smaller than zero and thus the one-sided tail probability has to be determined. An analogous differentiation is encountered in hypotheses testing [e.g., [75]]. If two *a priori *hypotheses are compared it cannot be predicted, which one is the preferred one by the data set and two-sided tail probabilities have to be determined. However, if the best tree is compared against an *a priori *hypothesis than the *a priori *hypothesis can never be better than the best tree and the one-sided tail probability has to be determined [75]. Furthermore, using the two-sided tail probabilities allows not only assessing excess conflict, but also excess support. This means, that the support of a node is mainly derived from one or only a few partitions. Thus, single gene artifacts within combined analyses can eventually be revealed. Finally, in comparison to one-sided tail probabilities two-sided ones are more conservative (i.e., indicate significance less often). Herein I used a significance level of 0.95. This means, that tails of 2.5% at each end of the distribution were used to determine significance. In case of a one-sided tail probability a tail of 5% at the left-hand side of the distribution would have been used for excess conflict. Thus, to convert the two-sided results into one-sided ones they have just to be divided by two.

In this study the potential of DRI, PABA and BS based approaches to detect conflict in data sets affected by paedomorphosis were compared analyzing compiled data of Wiens et al. [2] and Weisrock et al. [37]. Herein I will show that paedomorphosis affects the phylogenetic reconstruction of salamander phylogeny though the morphological partition provides only 14% of the parsimony-informative positions in the combined data set. Both strategies suggested by Wiens et al. [2] to ameliorate this effect are equally efficient, but overall the strategy to exclude paedomorphic character traits has to be favored over the strategy to recode paedomorphic taxa, because it deteriorates the phylogenetic signal in the morphological partition less severely. Concerning the molecular data, the rRNA partition seems to be too conserved to reliable reconstruct the salamander phylogeny while the mtDNA is too variable. The different approaches to detect incongruence are generally congruent to each other. However, the disagreement to the other methods is the lowest for approaches utilizing alteration measurements like NDI or PABA. Therefore, these approaches are preferable and with respect to computation time NDI outperforms PABA.

## Results

### Complete data set

**Figure 1:**
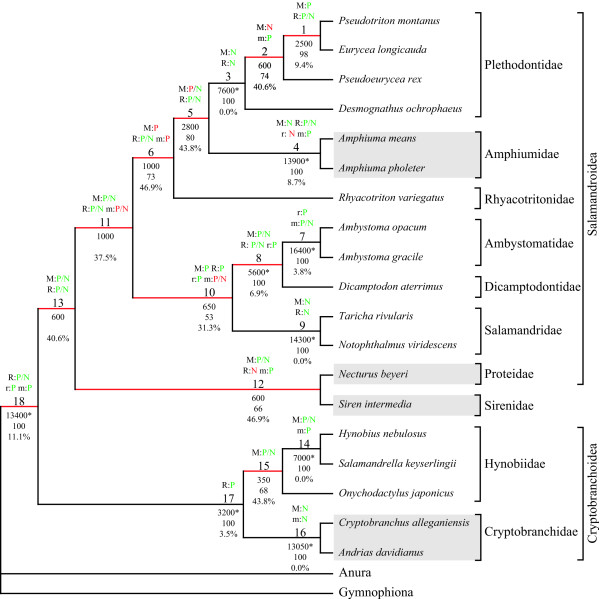
**Phylogenetic analysis of combined data and indicated conflicts**. Most parsimonious tree of the analysis of all data [Tree length (TL) = 858,325; Number of trees (NT) = 1]. Red branches are incongruent to trees obtained in analyses of all possible combinations of partitions (Figs. 3–5). The labels at the branches indicate the following from top to bottom: 1^st^) Significant contribution (green) or conflict (red) indicated by PABA (P) or NDI (N) for morphology (M), RAG1 (R), rRNA (r) or mtDNA (m); 2^nd^) Node labels; 3^rd^) BS values, with significant ones based on the Templeton [70] test indicated by an asterisk; 4^th^) BP values above 50; 5^th^) Degree of conflict indication. Paedomorphic taxa are highlighted with grey boxes.

The complete data set comprised 21 taxa and 6,427 characters in four partitions (morphology: 326; RAG1: 1,530; rRNA: 2,742; mtDNA: 1,829). The rRNA partition comprises sequence information of the small and the large subunit of the nuclear rRNAs and the mtDNA partition includes COI (subunit one of cytochrome *c *oxidase), ND1, ND2 (subunits one and two of NADH dehydrogenase), tRNA^Ala^, tRNA^Asn^, tRNA^Cys^, tRNA^Gln^, tRNA^Ile^, tRNA^Met^, tRNA^Trp^, tRNA^Tyr ^and the origin for light-strand replication. Of these characters 1,902 were parsimony informative and 482 uninformative. Thus, on average 105.7 informative characters per internal node could be expected. The phylogenetic reconstruction showed that families except for Hynobiidae are well supported by both BS and BP values (Fig. 1). Beyond the family level only the closer relationship of Ambystomatidae and Dicamptodontidae and the monophyly of Cryptobranchoidea (Hynobiidae plus paedomorphic Cryptobranchidae) as well as of Caudata are well supported. The paedomorphic Amphiumidae are placed as sister to Plethodontidae, whereas Sirenidae and Proteidae are sisters to each other and this group is then sister to the other Salamandroidea (i.e., Plethodontidae, Amphiumidae, Rhyacotritonidae, Ambystomatidae, Dicamptodontidae and Salamandridae) (Fig. 1).

Given the taxa in common, this tree (Fig. 1) is generally congruent with the combined analyses of Wiens et al. [2]. However, in their combined analyses *Desmognathus ochrophaeus *is more closely related to *Pseudotriton montanus *and *Eurycea longicauda *than is *Pseudoeurycea rex*. Furthermore, Proteidae and Sirenidae are not sister taxa to each other, but either Sirenidae occupy a more basal position [Fig. 7 in [2]] or Proteidae are sister to the clade comprising Ambystomatidae, Dicamptodontidae and Salamandridae [Fig. 8 in [2]]. On the other hand, this combined analysis (Fig. 1) and the combined analyses of Weisrock et al. [37] seem to be completely incongruent. However, a closer examination shows that the major difference is the placement of outgroups and thus the rooting of the tree. In their analyses the root is placed at the branch leading to paedomorphic Amphiumidae [Fig. 5 in [37]]. Furthermore, their analyses also do not support a sister group relationship of the paedomorphic taxa Sirenidae and Proteidae, but a paraphyletic assemblage of these with respect to the clade comprising Ambystomatidae, Dicamptodontidae and Salamandridae.

**Figure 2:**
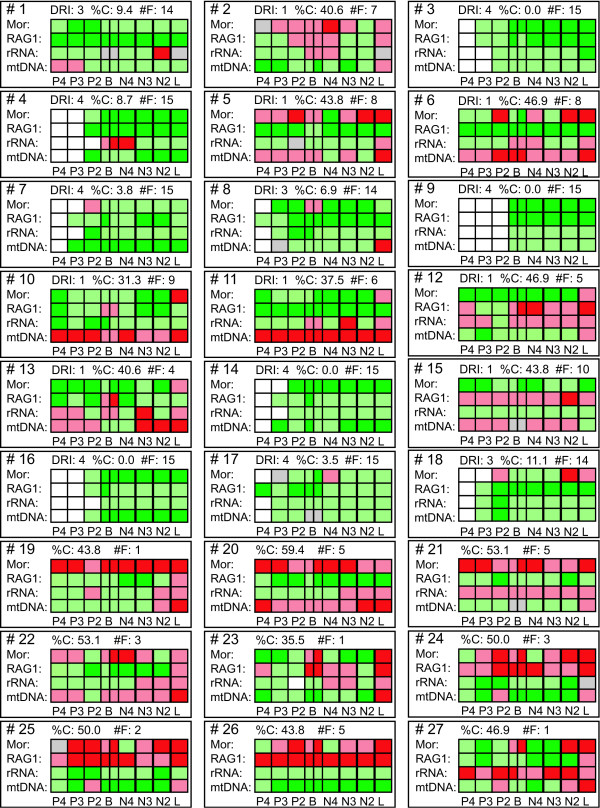
**Conflict or contribution at the first 27 nodes**. Negative (red boxes) or positive (green boxes) values at each node are indicated for each partition and approach: P4 = PABA_4_, P3 = PABA_3_, P2 = PABA_2_, B = PBS, N4 = NDI, N3 = PABSA_3_, N2 = PABSA_2_, L = LILD. Significant values are indicated by a stronger colour than non-significant ones (dark green vs. light green; dark red vs. light red). For PBS, the results of the Templeton [70] test are also indicated in the first half of the box. Grey boxes indicate a value of 0 and white boxes that the approach was not applicable due an alteration from maximal or minimal support (i.e., BP = 100 or 5) to maximal or minimal support (i.e., BP = 100 or 5). Furthermore, DRI, degree of conflict indication (%C) and number of times (#F) the node was found in the most parsimonious trees or consensus trees of the 15 different possible combinations of partitions (Figs. 1, 3-5) is given. The DRI is only given for the first 18 nodes corresponding to the tree of the analysis of all data (Fig. 1). At all other nodes DRI is 0.

**Figure 3:**
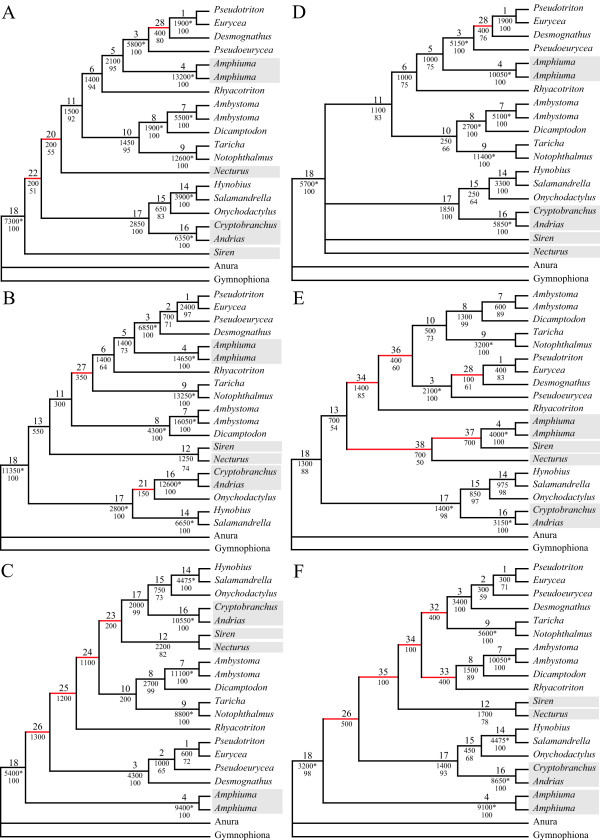
**Phylogenetic analyses of other combinations of partitions**. Most parsimonious trees or consensus trees of the analyses of other possible combinations of partitions. Strict consensus trees are given, when more than one most parsimonious tree was found. Red branches are incongruent to the tree obtained in the analysis of all data (Fig. 1). The labels at the branches of all trees indicate the following from top to bottom: 1^st^) Node labels; 2^nd^) BS values, with significant ones based on the Templeton [70] test indicated by an asterisk; 3^rd^) BP values above 50. Paedomorphic taxa are highlighted with grey boxes. A) Morphology+RAG1+rRNA [TL = 317,025; NT = 1]; B) Morphology+RAG1+ mtDNA [TL = 826,975; NT = 1]; C) Morphology+rRNA+mtDNA [TL = 657,125; NT = 1]; D) Morphology+RAG1 [TL = 286,325; NT = 2]; E) Morphology+rRNA [TL = 116,825; NT = 1]; F) Morphology+mtDNA [TL = 625,625; NT = 1].

**Figure 4:**
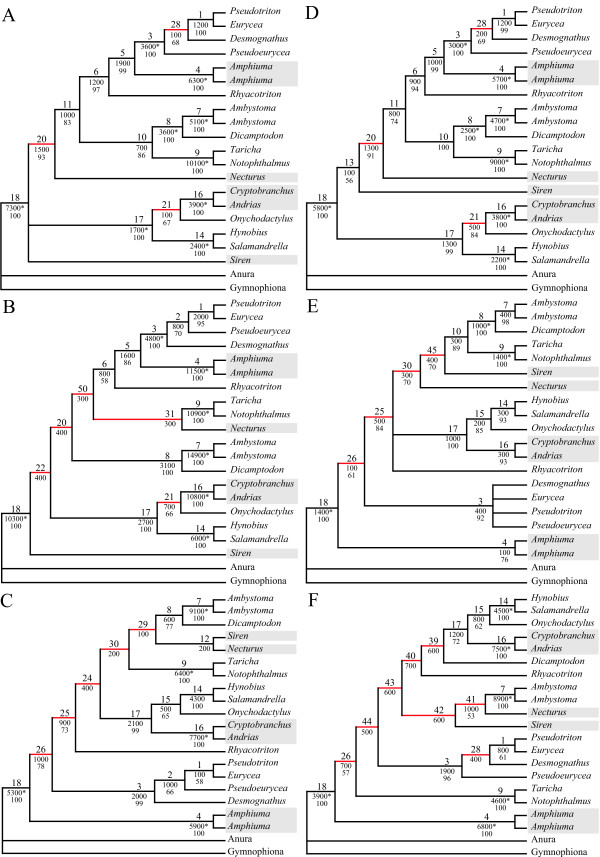
**Phylogenetic analyses of other combinations of partitions**. Most parsimonious trees or consensus trees of the analyses of other possible combinations of partitions. Strict consensus trees are given, when more than one most parsimonious tree was found. Red branches are incongruent to the tree obtained in the analysis of all data (Fig. 1). The labels at the branches of all trees indicate the following from top to bottom: 1^st^) Node labels; 2^nd^) BS values, with significant ones based on the Templeton [70] test indicated by an asterisk; 3^rd^) BP values above 50. Paedomorphic taxa are highlighted with grey boxes. A) RAG1+rRNA [TL = 231,500; NT = 2]; B) RAG1+mtDNA [TL = 741,400; NT = 1]; C) rRNA+ mtDNA [TL = 567,100; NT = 1]; D) RAG1 [TL = 200,400; NT = 1]; E) rRNA [TL = 29,700; NT = 4]; F) mtDNA [TL = 535,200; NT = 1].

**Figure 5:**
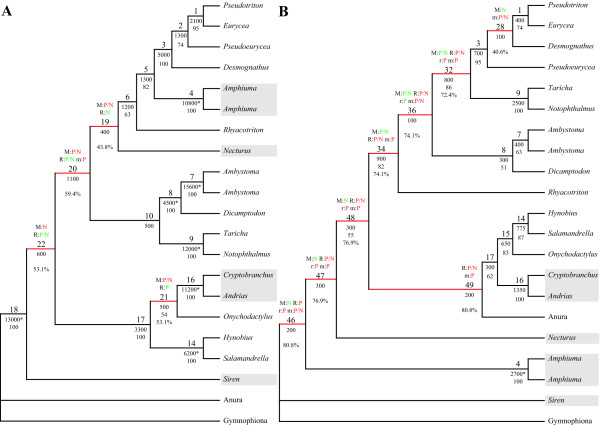
**Phylogenetic analyses of combined molecular data and only morphology with indicated conflicts for both**. Most parsimonious trees of the analyses of the combined molecular data (A) as well as only the morphological partition (B) [A: TL = 772,700; NT = 1; B: TL = 83,625; NT = 1]. Red branches are incongruent to the tree obtained in the analysis of all data (Fig. 1). The labels at the branches indicate the following from top to bottom: 1^st^) Significant contribution (green) or conflict (red) indicated by PABA (P) or NDI (N) for morphology (M), RAG1 (R), rRNA (r) or mtDNA (m); 2^nd^) Node labels; 3^rd^) BS values, with significant ones based on the Templeton [70] test indicated by an asterisk; 4^th^) BP values above 50; 5^th^) Degree of conflict indication. Paedomorphic taxa are highlighted with grey boxes.

**Figure 6:**
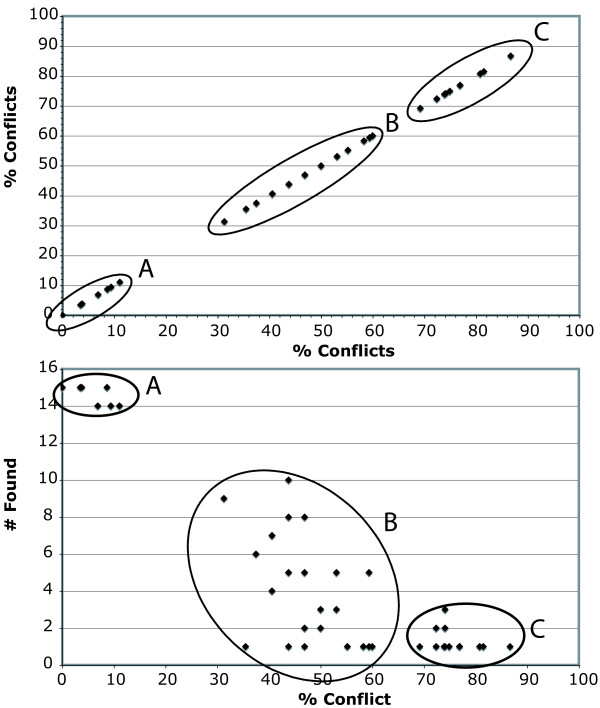
**Three different sets of nodes with different degrees of conflict indication**. The degree of conflict indication at each node is plotted against itself (upper graph) and the number of times the node was found in the in the most parsimonious trees or consensus trees of the 15 different possible combinations of partitions (lower graph). Circles indicate the three different groups A, B and C.

Only the nodes 3, 4, 7, 9, 14, 16 and 17 are consistently recovered in all possible combinations of partitions and thus have a DRI of 4 (Figs. 1, 2, 3, 4, 5). These nodes are all well supported and show no significant conflict due to any of the partitions except for node 4. At node 4 the rRNA partition exhibits a significant conflict based on the NDI and PBS approaches (Figs. 1, 2). Additionally, a few non-significant negative values are also obtained for others of these nodes (Fig. 2). However, all other approaches either result in positive values or are not applicable for these nodes. The degree of negative values and thus the indication of possible conflict ranges from 0–8.7% (Figs. 2 &6). This means, for example, that only 8.7% of the values are negative at node 4 (2 out of 23). Therefore, these values are most likely false negative ones.

The nodes 1, 8 and 18 are also within this low range of conflict indication (Figs. 2 &6). These three nodes are recovered in 14 out of the 15 trees (Figs. 1, 3, 4, 5), have a DRI of 3 and only one significant conflict is indicated for each (Fig. 2). The other negative values could have been obtained just by chance. Node 1 is not recovered by the individual rRNA partition analysis, because the rRNA partition cannot resolve ingroup relationships of Plethodontidae (Fig. 4E). In the mtDNA analysis (Fig. 4F) Dicamptodontidae are placed as sister to Cryptobranchoidea in contrast to node 8 and the morphological partition splits the outgroups apart (Fig. 5B). However, support for these placements is low in contrast to the well-supported alternatives in the combined analysis (Fig. 1). Overall, it seems safe to conclude that no partition really disagrees with the nodes belonging to group A in Fig. 6 (i.e., 1, 3, 4, 7–9, 14, and 16–18) and thus these nodes can be treated as conflict-free.

All other nodes of Fig. 1 exhibit a much higher degree of conflict indication ranging from 31.3% to 46.9%, which is accompanied by a DRI of only 1 (Fig. 2). Furthermore, the number of significant conflicts at these nodes is higher than at the other nodes of Fig. 1 (Fig. 2). For example, considering only PABA and NDI values significant conflict is indicated by morphology, RAG1 or mtDNA at five of seven nodes in contrast to one out of 11 (Fig. 1). The mtDNA partition exhibits strong and significant conflicts by nearly all methods at nodes 10 and 11 (Fig. 2). Furthermore, at these nodes as well as at nodes 5, 6, 13 and 15 negative values are consistently obtained by the different methods for the mtDNA partition. The same can be shown at nodes 12 and 15 for the RAG1 partition, although significant are only a few values. At node 12 also the rRNA partition nearly consistently results in negative values. The morphological partition indicates consistent and/or strong conflict at the nodes 2, 4 and 6. On the other hand, for the nodes 12 and 15, which in part place paedomorphic taxa, the major contribution of support is due to the morphological partition (Figs. 1, 2). Node 12 groups Sirenidae and Proteidae together and node 15, in concert with node 17, places Cryptobranchidae as sister to Hynobiidae (Fig. 1). Finally, these nodes (i.e., 2, 5, 6, 10–13, 15) are only found 3 to 9 times in the other 14 trees (Figs. 3, 4, 5) and none of these nodes is well supported (Fig. 1). Therefore, for each node at least one of the partitions is strongly disagreeing and thus they exhibit conflict due to that partition. Additional data or treatment of the data is necessary to either strengthen the support or minimize the conflict.

### Molecular versus morphological data

The combined molecular partitions have 1,636 parsimony informative and 430 uninformative positions. Thus, on average 90.9 informative characters per internal node could be expected. The most parsimonious tree (Fig. 5A) was nearly congruent with the tree obtained by the complete data set (Fig. 1). The paedomorphic Cryptobranchidae are a subtaxon of Hynobiidae and Sirenidae and Proteidae are not sister taxa to each other. Sirenidae branches off first within the Caudata and Proteidae are sister to the clade comprising Plethodontidae, Amphiumidae and Rhyacotritonidae. Not surprisingly the morphological partition is strongly disagreeing and in conflict with these four nodes different from the combined data set (i.e., 19–22; Figs. 2 &5A). The range of conflict indication at these nodes is overlapping with the range of the conflicting nodes of Fig. 1 (Figs. 2, 5A &6).

**Figure 7:**
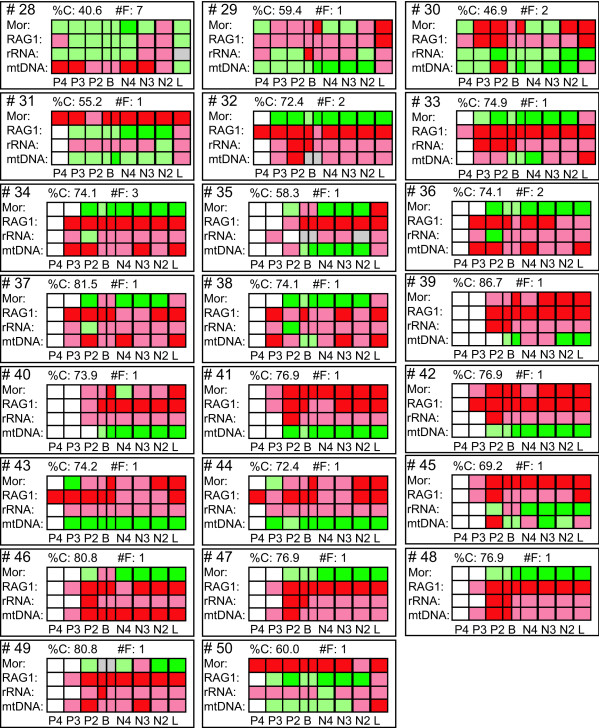
**Conflict or contribution at the remaining 23 nodes**. Negative (red boxes) or positive (green boxes) values at each node are indicated for each partition and approach: P4 = PABA_4_, P3 = PABA_3_, P2 = PABA_2_, B = PBS, N4 = NDI, N3 = PABSA_3_, N2 = PABSA_2_, L = LILD. Significant values are indicated by a stronger colour than non-significant ones (dark green vs. light green; dark red vs. light red). For PBS, the results of the Templeton [70] test are also indicated in the first half of the box. Grey boxes indicate a value of 0 and white boxes that the approach was not applicable due an alteration from maximal or minimal support (i.e., BP = 100 or 5) to maximal or minimal support (i.e., BP = 100 or 5). Furthermore, degree of conflict indication (%C) and number of times (#F) the node was found in the most parsimonious trees or consensus trees of the 15 different possible combinations of partitions (Figs. 1, 3–5) is given. At all nodes DRI is 0.

The morphological partition has 266 parsimony informative and 52 uninformative positions. Thus, on average 14.8 informative characters per internal could be expected. The most parsimonious tree (Fig. 5B) is incongruent to Fig. 1 in eight of 18 nodes. For example, the paedomorphic taxa Proteidae, Sirenidae and Amphiumidae are in a basal position and closely related to the outgroup, which is split apart. This is similar to the results of Wiens et al. [2] also showing a close relationship of these paedomorphic taxa analysing only the morphological data. The molecular data clearly and strongly reject these nodes except for node 28, which is the placement of *Desmognathus *closer to *Pseudotriton *and *Eurycea *(Figs. 5B &7). Only the mtDNA partition exhibits significant conflicts with this node, the other two molecular partitions show more positive values than negative ones. Furthermore, this node is also found in six other consensus trees and has with 40.6% a degree of conflict indication similar to the conflicting nodes of Figs. 1 and 5A. All other nodes (i.e., 32, 34, 36, 46–49) have a degree of above 72% (Figs. 5B &7).

Plotting the degree of conflict indication against itself as well as against the number of trees encompassing the particular node (Fig. 6) three distinct groups can be differentiated. Group A encloses all nodes with a degree of 0–11% and which are found in at least 14 consensus trees. These nodes can all be regarded as conflict-free (see above). Group B contains all nodes with a degree ranging from 30% to 60%. However, the number of times they were found is wide spread from one to 10, but a weak negative correlation between the number of times found and the degree of conflict indication can be seen. The third group C encompasses all nodes with a degree of conflict larger than 69% and the nodes are only found one to three times. These nodes are generally strongly and consistently rejected by three partitions indicating that their reconstruction is due to a single partition. In contrast to the nodes of group B, which are in need of further investigation, these nodes can be regarded single partition artefacts.

The nodes of group B are still or only recovered in combined analyses of all four partitions (i.e., 2, 5, 6, 10–13, 15; Fig. 1), of three partitions (i.e., 19–28; Figs. 3A–C, 5A) and of combinations of two molecular partitions (i.e., 29–31, 50; Fig. 4B&C). The nodes of group C are found either only in single partition analyses (i.e., 39–49; Figs. 4E&F, 5B) or in analyses of two partitions with one partition being the morphological one (i.e., 32–34, 36–38; Fig 3E&F). Node 35, which is found in the combined analysis of morphological and mtDNA data (Fig. 3F), is the only node of a bipartition analysis including morphological data that belongs to group B instead of C. All incongruent nodes of the morphology alone analysis belong to group C except for node 28 and thus can be regarded as nodes only supported or reconstructed by a single partition.

### Comparison of partitions

Comparing the degree of pairwise disagreements, that is one partition indicates a conflict or a contribution and the other one the contrary, reveals that the disagreement is the lowest for the pair RAG1 and rRNA (Table 1). On average the rRNA partition is slightly better than the RAG1 partition for all nodes and both being clearly better than mtDNA and morphological data. Considering only the nodes obtained in the analysis of all data (Fig. 1) RAG1 and rRNA are equally good, whereas the morphological partition exhibits lower average disagreement to the other partitions than the mtDNA partition. The results are similar for the nodes of the combined molecular data (Fig. 5A). Thus, RAG1 and rRNA show the lowest disagreement to each other as well as to the other two partitions. Considering all nodes morphology has the strongest disagreement to the other partitions, but regarding only the nodes of the two most inclusive analyses (Figs. 1 &5A) the mtDNA is disagreeing stronger than morphology.

**Table 1:** Disagreement of the different partitions to each other for three sets of nodes.

**All Nodes** **% Disagreement**		**Fig. 1** **% Disagreement**		**Fig. 5A** **% Disagreement**	
RAG1 ↔ rRNA	28.78	RAG1 ↔ rRNA	19.67	RAG1 ↔ rRNA	22.33
rRNA ↔ mtDNA	41.02	Mor ↔ rRNA	28.57	Mor ↔ rRNA	32.35
RAG1 ↔ mtDNA	43.30	Mor ↔ RAG1	29.37	Mor ↔ mtDNA	33.33
Mor ↔ rRNA	44.61	Mor ↔ mtDNA	36.07	RAG1 ↔ mtDNA	39.81
Mor ↔ RAG1	44.99	RAG1 ↔ mtDNA	37.80	rRNA ↔ mtDNA	41.58
Mor ↔ mtDNA	55.19	rRNA ↔ mtDNA	38.66	Mor ↔ RAG1	41.67
					
**Mean**		**Mean**		**Mean**	

rRNA	38.14	RAG1	28.94	rRNA	32.09
RAG1	39.02	rRNA	28.97	RAG1	34.60
mtDNA	46.51	Mor	31.33	Mor	35.78
Mor	48.26	mtDNA	37.51	mtDNA	38.24

% Disagreement indicates the percentage of the pairwise comparisons of partitions at which one of the partitions shows a negative value at the node and the approach used (PABA_4_, PABA_3_, PABA_2_, PBS, NDI, PABSA_3_, PABSA_2_, or LILD) while the other partition exhibits a positive value at the same node and the same method used. Mean shows the average disagreement each partition has to the other three partitions. Three sets of nodes were compared: 1^st^) all 50 nodes; 2^nd^) only the nodes of the analysis of all data (Fig. 1); 3^rd^) only the nodes of the analysis of the combined molecular data (Fig. 5A).

The mean contribution to the nodes of Fig. 1 is the strongest for the RAG1 partition, but the smallest for the rRNA partition with an average rank of 1 and 3.4, respectively (Table 2). The morphological partition is in between these two with an average rank of 2.9. This order is congruent with their number of parsimony informative positions ranging from 108 (rRNA) via 266 (morphology) to 533 (RAG1). However, the mtDNA partition does not fit into this order. With 995 it has the highest number of parsimony informative positions, but is only in 2^nd ^place for all BS based approaches and in 4^th ^for the PABA approach. Its average rank is similar to the morphological one (Table 2).

**Table 2:** Mean contribution of the different partitions to the nodes of Fig. 1 for each of the different approaches used as well as the corresponding rank.

	**Morphology**	**RAG1**	**rRNA**	**mtDNA**
**PABA_4_**	16 (2)	24 (1)	7 (3)	*-8 *(4)
**PABA_3_**	12 (2)	21 (1)	6 (3)	*-8 *(4)
**PABA_2_**	10 (3)	30 (1)	17 (2)	4 (4)
**PBS**	1197 (3)	2894 (1)	311 (4)	1472 (2)
**NDI**	1069 (3)	2743 (1)	475 (4)	1947 (2)
**PABSA_3_**	1135 (3)	2933 (1)	597 (4)	2163 (2)
**PABSA_2_**	837 (3)	2797 (1)	546 (4)	2031 (2)
**LILD**	71 (4)	2228 (1)	217 (3)	1444 (2)
				
**Average Rank**	2.9 (2 to 4)	1 (1 to 1)	3.4 (2 to 4)	2.8 (2 to 4)
				
**Parsimony informative positions**	266 (81.6%)	533 (34.8%)	108 (3.9%)	995 (54.4%)

After each mean contribution the rank is given in brackets. For example, for PABA_4 _the order of the mean contributions is first RAG1, second morphology, third rRNA and fourth mtDNA. The average rank for each partition as well as the range of ranks in brackets is also provided. The number of parsimony informative positions is given for each partition as well as their percentage of the total number of positions of the partition in brackets.

Finally, using the Wilcoxon signed rank test (WRST) it can be checked for any given set of nodes if the addition of a partition results in an increase or decrease of overall support despite the detection of some significant conflicts. Therefore, the value of the chosen approach (e.g., NDI or PBS) of each node of this set is ranked based on its absolute value, i.e. without its algebraic sign. Then the ranks of the positive values are summed up as well as the ranks of the negative values. Finally, it is determined if the rank sum of the positive and thus contributing values is larger or smaller than the rank sum of the negative ones and if this difference is significant. For example, if 10 of the 18 internal nodes had a positive value and all of them were larger in their absolute value than the other eight negative values the positive rank sum would be 135 and the negative one 36. Thus, it can be assessed if the inclusion of a partition is beneficial or detrimental over all considered nodes. Herein, I considered two sets of nodes. One set comprised the nodes of the tree of the analysis of all data (Fig. 1) and the other one the nodes of the tree of the combined molecular data (Fig. 5A). For nearly all BS based approaches the addition of any of the partitions is beneficial and most times this is significant at *p *≤ 0.05 (Table 3). However, the results of the PABA approach are not that clear cut. The addition of any partition as 2^nd ^is beneficial for all partitions. Whereas this holds also up for later additions as 3^rd ^or 4^th ^for RAG1 and rRNA, the morphological partition is becoming increasingly detrimental the later the addition takes place at the nodes of Fig. 5A. When added as 4^th ^this is significant at *p *≤ 0.05. As for the mtDNA, the same can be shown for both sets of nodes (Table 3). For the nodes of Fig. 1 the difference is already significant when added as 3^rd ^partition.

**Table 3:** WRST results, if the contribution of a partition to the nodes of either Fig. 1 or Fig. 5A is beneficial or detrimental.

	**Morphology**							
	**PABA_4_**	**PABA_3_**	**PABA_2_**	**PBS**	**NDI**	**PABSA_3_**	**PABSA_2_**	**LILD**
**Fig. 1**	< 0.00001	< 0.00001	< 0.00001	< 0.00001	< 0.00001	< 0.00001	< 0.00001	0.19602
**Fig. 5A**	***0.00556***	*0.07690*	< 0.00001	< 0.00001	< 0.00001	< 0.00001	< 0.00001	*0.07926*
	**RAG1**							
**Fig. 1**	< 0.00001	< 0.00001	< 0.00001	< 0.00001	< 0.00001	< 0.00001	< 0.00001	< 0.00001
**Fig. 5A**	< 0.00001	< 0.00001	< 0.00001	< 0.00001	< 0.00001	< 0.00001	< 0.00001	< 0.00001
	**rRNA**							
**Fig. 1**	< 0.00001	< 0.00001	< 0.00001	< 0.00001	< 0.00001	< 0.00001	< 0.00001	< 0.00001
**Fig. 5A**	< 0.00001	< 0.00001	< 0.00001	< 0.00001	< 0.00001	< 0.00001	< 0.00001	0.00001
	**mtDNA**							
**Fig. 1**	***0.00768***	***0.03220***	0.00842	< 0.00001	< 0.00001	< 0.00001	< 0.00001	< 0.00001
**Fig. 5A**	***0.00446***	*0.16574*	< 0.00001	< 0.00001	< 0.00001	< 0.00001	< 0.00001	0.00002

*p *values of the WRST are given for each partition, each approach and the two sets of nodes. Italic values indicate that the negative rank sum was larger than the positive rank sum and thus the contribution of the partition is detrimental to this set of nodes given the used approach. If they are also bold this difference was significant.

On the other hand, ILD tests show that the morphological and RAG1 partitions are incongruent to each other as well as to the other two partitions or any combination of partitions (Table 4). The mtDNA partition results only in the pairwise comparison with the rRNA partition in a non-significant *p *value, in all other comparisons this value is below 0.05. In contrast to these three partitions, the rRNA partition exhibits incongruence only in the pairwise comparisons to either the morphological or the RAG1 partition.

**Table 4:** ILD results of the comparison of each partition against all combinations of the other partitions.

**against**	**Morphology**	**RAG1**	**rRNA**	**mtDNA**
**Morphology**	-	**0.001**	**0.001**	**0.001**
**RAG1**	**0.001**	-	**0.016**	**0.009**
**rRNA**	**0.001**	**0.016**	-	0.955
**mtDNA**	**0.001**	**0.009**	0.955	-
**Morphology+RAG1**	-	-	0.204	**0.005**
**Morphology+rRNA**	-	**0.001**	-	**0.004**
**Morphology+mtDNA**	-	**0.005**	0.263	-
**RAG1+rRNA**	**0.001**	-	-	**0.003**
**RAG1+mtDNA**	**0.001**	-	0.514	-
**rRNA+mtDNA**	**0.001**	**0.001**	-	-
**All three others**	**0.001**	**0.002**	0.339	**0.001**

Significant *p *values of the ILD test are in bold. -- = comparison not applicable

### Strategies ameliorating effects of paedomorphosis

Wiens et al. [2] suggested two strategies to ameliorate negative effects of paedomorphosis on phylogenetic reconstructions using morphological data. The first one (S1) is to code characters of the adult morphology of non-paedomorphic species as unknown in paedomorphic species. The other one (S2) is to remove specifically characters from the data set, which are supposedly affected by paedomorphosis. Additionally, it is also possible to combine these two strategies in one (S12) [2]. In analyses based on all four partitions each of these three strategies resulted in the same topology, which is identical with the topology of the analysis of the combined molecular data (Fig. 5A). Each strategy minimizes the conflict at the nodes 19 to 22 induced by the morphological partition, i.e. the values increase (e.g., from -800 to 400 for the LILD approach, the strategy S12 and node 20). The only exception is the PBS value at node 21, which decreases using strategy S2 from -650 to -950.

Strategy S1 codes 27.8% of the morphological data as unknown and thus effectively deletes them. Strategy S2 deletes 9.2% of the morphological data and the combination of both strategies S12 results in a deletion of 34.4%. Thus, though the three strategies minimize the conflict at nodes 19 to 22 due to morphology the effect at the remaining nodes of Fig. 5A has also to be assessed to show whether the strategies are overall beneficial or detrimental. Using the WRST it can be tested if the rank sum of the positive changes, i.e. an increase in the values, is larger or smaller than the rank sum of the negative values as well as if this difference is significant for all nodes of Fig. 5A. Therefore, the difference of the values after and before the treatment at a node is calculated (e.g., 400-(-800) = 1200 for the LILD approach, the strategy S12 and node 20). These differences are ranked based on their absolute values and the ranks of the positive values are summed up as well as the ranks of the negative values. All three strategies are overall significantly detrimental to PBS values (Table 5). On the other hand, strategy S2 is significantly beneficial in all other approaches. Furthermore, the average degree of disagreement of the treated morphological partition to the other partitions is only 31.14% and thus is 4.64% smaller than for the untreated morphological partition (Tables 1 &5). For strategy S1 and S12, the results are not so unequivocal. PABA and LILD generally exhibit a larger positive rank sum, but only the differences of strategy S12 and both PABA as 4^th ^and as 3^rd ^are significant. In contrast, NDI and PABSA changes are overall detrimental, except for NDI and strategy S1. In three cases the changes are significant (Table 5). Additionally, for both the average degree of disagreement increases by 2.50% and 4.43%, respectively.

**Table 5:** WRST results of the overall effects of the different strategies to ameliorate the negative impact of paedomorphosis on the phylogenetic reconstruction based on the nodes of Fig. 5A.

	**Strategy**		
	**S1**	**S2**	**S12**
**PABA_4_**	0.00252	**<0.00001**	**0.00019**
**PABA_3_**	0.07684	**<0.00001**	**0.00319**
**PABA_2_**	0.00128	**<0.00001**	0.02480
**PBS**	***<0.00001***	***<0.00001***	***<0.00001***
**NDI**	0.85342	**<0.00001**	***0.00558***
**PABSA_3_**	***<0.00001***	**0.00064**	***<0.00001***
**PABSA_2_**	*0.16582*	**<0.00001**	*0.51783*
**LILD**	0.76970	**<0.00001**	0.53493
			
**Change in mean disagreement**	*+2.50*	-4.64	*+4.43*

Significant *p *values are in bold. Italic values indicate that the negative rank sum was larger than the positive rank sum and thus the strategy is overall detrimental to this set of nodes given the used approach (PABA_4_, PABA_3_, PABA_2_, PBS, NDI, PABSA_3_, PABSA_2_, or LILD). Furthermore, the change in the mean disagreement of the morphological partition to all other partitions at these nodes is also shown (Table 1). S1 = code adult morphology of paedomorphic taxa as unknown; S2 = exclude paedomorphic character traits; S12 = combination of S1 and S2.

### Comparison of different approaches

**Figure 8:**
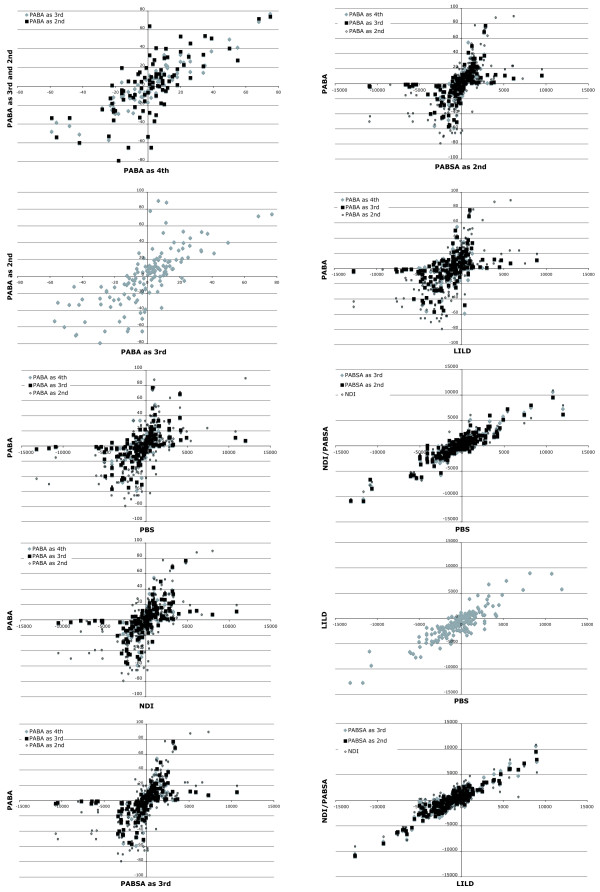
**Pairwise comparisons of the different conflict detecting approaches to each other**. The values of each approach (PABA_4_, PABA_3_, PABA_3_, PBS, NDI, PABSA_3_, PABSA_2_, or LILD) are plotted against each other approach to analyse the correlations of their values. The plots of the pairwise comparisons of NDI, PABSA_3 _and PABSA_2 _to each other are shown in Fig. 9.

**Figure 9:**
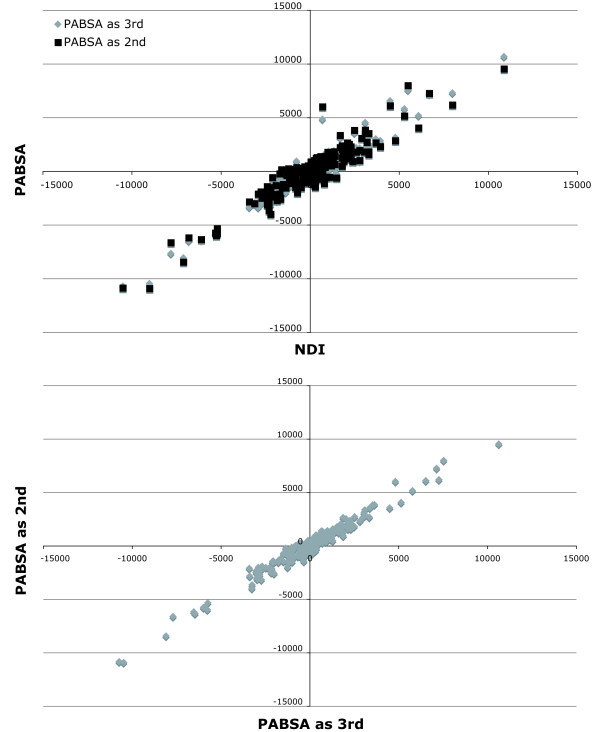
**Pairwise comparisons of the NDI/PABSA approaches to each other**. The values of each NDI/PABSA approach (NDI, PABSA_3 _and PABSA_2_) are plotted against each other to analyse the correlations of their values. The other comparisons are shown in Fig. 8.

All approaches tested herein to detect conflict show a positive correlation to each other (Figs. 8, 9). The correlation within BS based approaches and PABA is stronger than between these approaches. However, this is not surprising due to the fact that the amplitude of alteration is different though the algebraic sign is the same. Furthermore, in comparisons with PBS or LILD the distribution is shifted slightly to more negative PBS or LILD values.

In pairwise comparisons of the approaches both approaches can have either the same algebraic sign or different ones. Table 6 shows the percentage of pairs, in which one approach has a negative and thus a conflicting value for a node and partition while the other one has a positive contributing one. On average LILD indicates in 24.84% of the comparisons a conflict, while the other approach indicates a positive value. The PBS approach has the 2^nd ^highest mean with 11.27%. PABA and NDI/PABSA range from 5 to nearly 8%. In both, addition as 2^nd ^has the highest percentage (Table 6).

**Table 6:** Percentage disagreement of the different approaches to each other at all nodes.

**Negative values (conflict)**	**Positive values (contribution)**								**Mean**
	**PABA**_4_	**PABA**_3_	**PABA**_2_	**PBS**	**NDI**	**PABSA**_3_	**PABSA**_2_	**LILD**	
**PABA_4_**		4.30	9.78	3.23	8.60	1.08	9.68	4.30	5.85
		*0.00*	*0.00*	*0.00*	*2.70*	*0.00*	*0.00*	*1.82*	*0.65*
**PABA_3_**	4.30		7.33	3.27	8.50	3.27	5.88	2.61	5.02
	*2.22*		*2.20*	*1.18*	*5.36*	*1.22*	*0.00*	*1.10*	*1.90*
**PABA_2_**	14.13	7.33		6.35	10.58	7.41	5.82	1.59	7.60
	*11.54*	*6.59*		*4.03*	*8.87*	*5.43*	*3.94*	*0.00*	*5.77*
**PBS**	15.05	11.11	13.76		12.00	9.00	13.00	5.00	11.27
	*15.38*	*9.41*	*7.26*		*13.51*	*10.26*	*11.86*	*4.96*	*10.38*
**NDI**	5.38	6.54	6.88	2.50		3.50	7.50	2.50	4.97
	*0.00*	*3.57*	*4.03*	*2.70*		*1.85*	*4.39*	*1.61*	*2.59*
**PABSA_3_**	7.53	7.19	8.99	4.00	8.00		6.50	2.50	6.39
	*2.22*	*2.44*	*3.10*	*1.71*	*4.63*		*3.64*	*2.34*	*2.87*
**PABSA_2_**	13.98	8.50	6.88	6.50	10.50	5.00		2.00	7.62
	*7.69*	*4.60*	*2.36*	*2.54*	*8.77*	*3.64*		*0.00*	*4.23*
**LILD**	38.71	27.45	22.75	18.00	25.00	20.50	21.50		24.84
	*27.27*	*21.98*	*14.93*	*18.18*	*27.42*	*22.66*	*19.84*		*21.75*

The percentage is relative to the total number of data pairs in pairwise comparisons of the approaches. The upper values were obtained considering all pairs and the lower value considering only pairs, which had at least one significant value. For example, in the comparison of PABA_4 _to PABA_3 _in 4.3% of the data pairs PABA_4 _had a negative value while PABA_3 _had a positive one, when all pairs were considered, and in 0% of the data pairs, when only pairs with at least one significant value were considered. For PBS, the Templeton [70] test results were not considered.

Considering only pairs, in which at least one of the two values is significant, clearly decreases the mean values as well as the values of the individual pairwise comparisons (except for a few instances) (Table 6). LILD and PBS still exhibit the highest mean values with 21.75% and 10.38%, respectively. PABA and NDI/PABSA range from 0.65% to 5.77% and from 2.59% to 4.23%, respectively. In both, the mean values decrease with increasing order of addition. Especially strong is the decrease in the PABA approach, when a partition is added as 4^th^. The percentage decreases from 5.85% to only 0.65%. In five out seven individual comparisons the percentage is even 0.00%. This means that there is no negative value, which is accompanied by a positive value in the other approach of the pairwise comparison.

## Discussion

### Paedomorphosis, morphology and conflict

The addition of morphological data to molecular data changed the phylogenetic positions of three of the four paedomorphic taxa included in this study. Cryptobranchidae moved to a more basal position within Cryptobranchoidea and Sirenidae and Proteidae are grouped together. Additionally, the morphological partition exhibits strong conflict with nodes separating Proteidae and Sirenidae in the combined molecular analysis. The same can be shown for the node placing Cryptobranchidae as a subtaxon of Hynobiidae. Thus, morphology can exhibit even in the combination with large molecular data sets strong misleading effects due to paedomorphosis [2,31-35]. For example, the molecular data recover the monophyly of Salamandroidea, which also comprises Proteidae, but not Sirenidae. Sirenidae and Cryptobranchoidea reproduce by external fertilisation [76]. Salamandroidea are characterized by internal fertilisation, which requires a specific complex of male and female cloacal glands, life history and behaviour [77]. This complex has never been lost within any of these families. This indicates that it is evolutionary highly 'burdened' in the sense of Riedl [78] and Donoghue [79] and is unlikely to be secondarily lost [37]. Still the morphological data render Salamandroidea paraphyletic.

Furthermore, all strategies to minimize the negative impact of paedomorphic taxa and/or character traits resulted in trees identical to the molecular one as well as reduced conflicts at the corresponding nodes. Thus, morphological data of paedomorphic taxa and/or character traits can mislead the phylogenetic reconstruction, but this impact can be ameliorated by different strategies employed herein. On the other hand, in the analyses of Wiens et al. [2] no major difference could be observed between the tree obtained from their untreated combined data set and the trees obtained from their treated combined data sets. Wiens et al. [2] used the same strategies to treat their combined data set as employed herein. Additionally, their obtained trees for the combined data were relatively similar to the trees obtained only from the molecular data. Thus, in the analyses of Wiens et al. [2] the negative impact of morphological data of paedomorphic taxa on the phylogenetic reconstruction using the combined data set was much less influential than in the analyses presented herein. The analyses of Wiens et al. [2] comprised 50% more paedomorphic and non-paedomorphic taxa than this analysis. Therefore, not only the strategies analyzed herein may reduce the effect of paedomorphosis on combined analyses, but also an increased taxon sampling.

Based on the results presented herein the strategy to exclude paedomorphic character traits is to be favoured over the strategy to recode paedomorphic taxa or the combination of both. Due to possible difficulties to securely determine paedomorphic character traits Wiens et al. [2] favoured the strategy to recode taxa. Furthermore, Wiens et al. [2] showed that other factors like synapomorphies of adult characters in non-paedomorphic taxa and convergent evolution have an impact on the phylogenetic assessment of paedomorphic taxa. Both strategies were able to ameliorate the effects of paedomorphosis on morphological data sets herein. However, based on the WRST results the strategy to just exclude supposedly paedomorphic character traits seems to be also overall beneficial. The other strategy and the combination of both are overall either beneficial or detrimental depending on the method. The latter means that though the conflict at the nodes affected by paedomorphosis is minimized the support at the other nodes due to the morphological partition is strongly reduced as well. The number of effectively deleted characters is at least three times higher in these two strategies than in the strategy to exclude traits. Thus, not only conflicting characters are deleted with a higher probability, but also supporting characters.

The analyses revealed that there is substantial disagreement concerning the position of paedomorphic Cryptobranchidae. Monophyly of Cryptobranchoidea comprising Hynobiidae and Cryptobranchidae is well supported by both BP and BS values. However, the position of Cryptobranchidae within Cryptobranchoidea is controversial. Morphology and rRNA contribute to the placement as sister to Hynobiidae, whereas RAG1 and mtDNA contribute stronger to a derived position within Hynobiidae. Different strategies ameliorating the negative impact of paedomorphosis show that the contribution for the more basal placement of Cryptobranchidae by morphology is in part due to paedomorphic character traits. Interestingly, the combined analyses of both Wiens et al. [2] and Weisrock et al. [37] recovered monophyletic Hynobiidae, whereas the combined molecular analyses of Wiens et al. [2] found their paraphyly. Therefore, to securely resolve the phylogenetic position of Cryptobranchidae within Cryptobranchoidea more molecular data are needed.

### Molecular partitions and conflict

Though the morphological partition is influenced by paedomorphosis the molecular partitions show substantial conflicts as well. RAG1 and rRNA are disagreeing the least with each other and with other partitions, though the topologies obtained in the individual analyses are very different [Fig. 4D&E and see also [2,37]]. Taxonomic congruence approaches would have indicated that there is substantial disagreement between the two partitions and thus would have given misleading results to not combine the two. The addition of both partitions to the data set is always beneficial based on WRST results and only one significant conflict based on PABA or NDI can be detected for each at the nodes of the two most inclusive data sets, the complete one and the complete molecular one. However, the contribution of RAG1 to the analyses is much stronger than that of rRNA. This is not surprising given that RAG1 has nearly five times more parsimony-informative positions. On the other hand, the rRNA partition has nearly twice the size. Thus, with respect to salamander phylogeny the rRNA genes might actually be too conserved given that only 3.9% of positions are informative for parsimony reconstructions. If genes are too conserved they might not be able to resolve nodes, especially short internal ones as in the case of the salamander phylogeny [37], because not enough phylogenetic signal is picked up to robustly resolve the phylogeny [80,81]. This is also reflected in the ILD results. While the other three partitions are at odds which each other, the rRNA partition shows congruence to nearly all possible combinations of partitions.

In contrast, the mtDNA partition exhibits significant conflicts based on PABA or NDI at three nodes of the two most inclusive data sets and disagrees the strongest with the other partitions on the nodes obtained by these data sets. The addition of mtDNA data as third or fourth is detrimental for bootstrap support. Upon addition BP values decline more often and stronger than they increase. This is congruent with the average contribution, which is slightly negative. The contribution of mtDNA is always smaller than that of RAG1 though it has nearly twice as much parsimony informative positions in only 1.2 times as much positions. Using simulation studies Weisrock et al. [37] showed that mtDNA might lack the potential to robustly resolve short internal branches deep in the salamander phylogeny. Two taxa splitting 35 million years ago must have had an ancestral lineage of at least 5 to 9 million years to be robustly reconstructed by Bayesian inference or parsimony, respectively, using mtDNA data [37]. However, fossil records for some extant families such as Cryptobranchidae date back to the early Cretaceous [e.g., [82]] while the earliest salamanders are found in the late Jurassic [36]. Thus, salamander presumably showed a rapid radiation resulting in short internal branches and long terminal ones [37,83]. Such phylogenies are hard to resolve [e.g., [80,81,84-86]]. Furthermore, though mtDNA adds twice as much parsimony informative positions it is not able to overwhelm the phylogenetic signal of RAG1 in combined analyses. For example, the outgroup taxa are never placed in such cases close to the Amphiumidae (Figs. 1, 3B, 4B, 5A) as they are in the mtDNA alone analysis [Fig. 4F and [37]]. A consequence of the latter is also that Salamandroidea are not monophyletic as they are for example in the RAG1 alone analysis (Fig. 4D). On the other hand, BS based approaches and PABA_2 _show that the addition of the mtDNA is beneficial to nodal support values. This is due to more recent branching events in the salamander phylogeny to which the mtDNA contributes positively. This is in agreement with the results of Weisrock et al. [37] and indicates that mtDNA can add substantial support to more recent evolutionary events. Therefore, while rRNA seems to be too conserved for the reconstruction of salamander phylogeny mtDNA seems to be too variable for nodes deep in the salamander phylogeny. However, to robustly resolve salamander phylogeny more molecular data are advisable, because they are in contrast to morphological data not affected by paedomorphosis [e.g., [22]]. Future studies concerning salamander phylogeny should concentrate on genes with properties similar to the RAG1 gene, which seems to be the most potential gene in this study to resolve the deep salamander phylogeny.

### Wilcoxon signed rank tests and partition congruence

Herein I presented a new approach to determine congruence of partitions based on node-by-node values of different methods. This approach utilized the Wilcoxon Signed Rank Test (WRST) to test whether more negative or positive values are obtained for a set of nodes and also takes into account the magnitude of these values. Concerning WRST results there seems to be a discrepancy between PABA_3&4 _and both the BS based approaches and PABA_2_, if the addition of the morphological or mtDNA partitions is detrimental or beneficial, respectively. This discrepancy is not contradictory. The WRST is conservative in the indication of a detrimental effect and thus in its advice against merging the partitions. Therefore, the negative rank sum has to be larger than the positive rank sum. To achieve this either more than half of the nodes exhibit slight conflicts without the positive ones showing strong contributions or less than half, but several of the nodes have very strong conflicts and the other nodes provide only small positive contributions. To be significant the difference between the rank sums has to be even more pronounced. With increasing numbers of partitions more and more nodes are maximally supported by a BP value of 100 and cannot be increased anymore if a new partition is added. Thus, the PABA approach is not applicable at these nodes anymore and the nodes will not be counted in the WRST. On the other hand, conflicts induced at nodes by a partition might still be detected as long as the conflict is able to decrease the BP value below 100. As a consequence, the ratio of the nodes with negative values to the ones with positive values is shifted to the negative ones with increasing order of addition. In contrast, BS based approaches are not hampered by such an upper limit and thus the ratio of these nodes will not be altered. However, this is not necessarily a disadvantage for the PABA approach. Due to the PABA approach conflicts, which are persistent in a partition and thus might cause strong incongruence, can be more easily detected by means of WRST. Thus, while the mtDNA and morphology is contributing to several nodes, which are also supported by other partitions, both show also significant incongruence at some nodes. On the other hand, the ILD test [41] seem to be too sensitive to incongruence at a few internal nodes by indicating that except for the very slowly evolving rRNA partition all partitions are at odds with each other. For the more detailed discussion of the pros and cons of ILD see the Background section and literature cited therein.

### Comparison of conflict detecting approaches

The results presented herein indicate that approaches using alteration estimations, NDI/PABSA and PABA, are more conservative in the indication of conflict than the approaches only analyzing individual partitions such as PBS and LILD. The latter ones indicate more often a conflict while other approaches indicate a positive contribution. Alteration approaches such as NDI assess not only the conflict and support within individual partitions, but also the support or conflict the addition of a partition brings out in the other partitions, which is also known as hidden support [40]. Thus, alteration methods also take into account the reciprocal effects of partitions to each other.

In both approaches the addition as 2^nd ^and thus the first possible addition is the least conservative one. Combining partitions might increase the weak phylogenetic signal in each individual partition while the phylogenetic signal-like pattern due to homoplasy remains constant or even decreases [e.g. [40,56]]. Thus, with only two partitions the influence of homoplasy in each partition can still be strong enough to indicate conflict with one approach and contribution with another one, though either the conflict or the contribution is weak. Or in other words, the full hidden support is not revealed yet for both partitions. With increasing amount of data in one of the partitions (i.e., later addition of the other partition) the reciprocal influence of the partitions to each other is more easily revealed and thus PABA and NDI/PABSA, which are able to assess this influence, are increasingly conservative with later additions. Additionally, for PABA sample size may also be in part accountable for this trend. With increasing number of addition more nodes are either maximally or minimally supported (i.e., 100% or 5%, respectively) and are not altered anymore. Thus, the sample sizes decrease and the chance to disagree with other approaches. Weak conflicts or contributions cannot be revealed any longer, because they are not able to overwhelm the signal in the other partitions. This might also in part explain the very low percentages of disagreement obtained for PABA_4 _considering only significant values of conflict or contribution. However, PABA and NDI/PABSA results are generally congruent to each other. Thus, a full-blown PABA or NDI/PABSA might not always be needed to reveal the conflict at nodes, but only the last possible addition (e.g., as 4^th^). Especially for NDI/PABSA only NDI seems to be necessary given that NDI is not affected as PABA by already maximally supported nodes. In PABA these nodes are often not applicable anymore for the detection of conflict or support and lower orders of additions are needed, if effects of partitions on BP support of these nodes shall be shown. Additionally, NDI is less computational intensive than PABA (see below). On the other hand and as discussed above, due to the 'removal' of maximally supported nodes PABA is more efficient to show persistent conflict using WRST.

LILD is less conservative than PBS. LILD assesses the contribution of a partition to a node based on tree reconstructions of the individual partition. Therefore, LILD suffers from the same problems as taxonomic congruence approaches [e.g., [40,53,56-58]]. They cannot recognize hidden support in the data sets due to overwhelming signal-like noise. In concatenated analyses the hidden support might be revealed, because the noisy positions cancel each other out while the signal increases [40]. For PBS, the individual partition is mapped on trees obtained by the complete data sets. Therefore, the tree search takes the hidden support into account whereas the calculation of PBS is based on the individual partition. Gatesy et al. [40] pointed out that PBS is not able to assess the contribution a partition reveals in the other partitions, but the contribution the partition has in the simultaneous analysis. PBS is the sum of the BS value of the partition (= LILD value) and the hidden support/conflict of the partition for that node [40]. Thus, PBS is in contrast to the LILD test able to show some of the hidden support in the data set, but not all as in alteration approaches.

However, in revealing the degree of conflict at specific nodes as well as disagreement between partitions the combination of all approaches were most powerful. For example, due to 20 to 32 conflict assessments at each node an overall degree of conflict for each node could be obtained. This procedure revealed three distinct groups of nodes. The first group could be regarded as undisputed by all partitions and thus regarded as unequivocal supported by them. This support is equivalent to a taxonomic congruence approach in that these nodes gain positive contribution from different partitions and methods over and over again and thus gather increasing verification [e.g., [51,87-90]]. In contrast the third group contained nodes, which were opposed by the vast majority of the tests and are usually accompanied by low nodal support in analyses, which recovered the node. Thus, these nodes can be seen as falsified in a Popperian sense [89-92]. The second group contains all nodes, which are equivocally supported. These nodes gather support by some of the partitions and are in conflict with others. Therefore, these nodes are in need of further investigations and eventually data. Based on the definition of Grant and Kluge [93] and their provided example of unweighted parsimony I would regard the procedure presented herein as scientific employing empirical tests. For unweighted parsimony the "propositions of relative recency of common ancestry [i.e., the nodes herein] are tested according to the congruence/incongruence of available [empirical] character evidence" [93]. The methods invoked herein similarly tested the congruence or incongruence of nodes using the available character evidence, which was grouped into different partitions. On the other hand, WRST and the randomization of partitions can be seen as heuristic methods in the sense of Grant and Kluge [93] to investigate the strength of the obtained values.

Although the application of several different approaches is preferable to gain a more thorough overview of support and conflict this might not be always feasible due to computation limits (see below). In this case the number of approaches should be reduced in such a way, that the number of tree searches is reduced, but also that different kinds of nodal support measurements are exploited to check whether support and conflict are similar for these as in this study. For example, one could use only the addition as last partition for PABA (e.g., PABA_4 _herein) in combination with NDI. Furthermore, if NDI is done the tree searches for PBS are already conducted as well. Therefore, if NDI is chosen, PBS can be easily obtained as well with not much additional computation time. If the last addition in PABA is not applicable at all nodes or most nodes, PABA_2 _or the next lower addition (e.g., PABA_3 _herein) could be used instead because of all alternative additions these two require the fewest tree searches. Especially in analyses with several partitions, e.g. more than 10, PABA_2 _might be more suitable as the first choice to effectively balance between the number of tree searches and the number of results actually obtained.

### Computation time and likelihood methods

Assuming that computation time of a tree search is roughly the same regardless if it is an unconstrained, constrained, anti-constrained or single bootstrap replicate search, the computation time to calculate BS based and PABA values is proportional to the number of tree searches to be conducted albeit some time has to be added for actual calculations of the values. For PBS the number of trees searches depends on the number of nodes to be analyzed. These are usually at least the nodes of the best tree of all data. Thus, one tree search is necessary to find the best tree as well as anti-constrained searches for each internal node. The number of internal nodes is the number of taxa minus three. However, for each additional node of interest not obtained in the best tree constrained searches are also needed:

*S*_*PBS *_= *t *- 2 + *any additional node*

with *S *being the number of tree searches and *t *the number of taxa.

For LILD and NDI equation 4 has to be multiplied by the number of partitions *P*, because each node or its best alternative has to be searched for in either each individual partition or each data set minus that partition, and additionally the number of tree searches necessary for the complete data set (Equation 4) has to be added for NDI:

*S*_*LILD *_= P * *S*_*PBS*_

and

*S*_*NDI *_= P * *S*_*PBS *_+ *S*_*PBS *_

For DRI, NDI/PABSA and PABA the number of all possible combinations of partitions has to be known:

∑k=1P(Pk)=∑k=1PP!k!(P−k)!≡2P−1
 MathType@MTEF@5@5@+=feaafiart1ev1aaatCvAUfKttLearuWrP9MDH5MBPbIqV92AaeXatLxBI9gBaebbnrfifHhDYfgasaacPC6xNi=xI8qiVKYPFjYdHaVhbbf9v8qqaqFr0xc9vqFj0dXdbba91qpepeI8k8fiI+fsY=rqGqVepae9pg0db9vqaiVgFr0xfr=xfr=xc9adbaqaaeGacaGaaiaabeqaaeqabiWaaaGcbaWaaabCaeaadaqadaqaauaabeqaceaaaeaacqWGqbauaeaacqWGRbWAaaaacaGLOaGaayzkaaGaeyypa0ZaaabCaeaadaWcaaqaaiabdcfaqjabcgcaHaqaaiabdUgaRjabcgcaHiabcIcaOiabdcfaqjabgkHiTiabdUgaRjabcMcaPiabcgcaHaaaaSqaaiabdUgaRjabg2da9iabigdaXaqaaiabdcfaqbqdcqGHris5aOGaeyyyIORaeGOmaiZaaWbaaSqabeaacqWGqbauaaaabaGaem4AaSMaeyypa0JaeGymaedabaGaemiuaafaniabggHiLdGccqGHsislcqaIXaqmaaa@4ED9@

For DRI less than these might be necessary in a successive approach as done by Gatesy et al. [40]. For NDI/PABSA equation 7 has to be multiplied with equation 4 and for PABA with the number of bootstrap replicates *r*:

*S*_*NDI*/*PABSA *_= (2^*P *^- 1) * *S*_*PBS *_,

*S*_*PABA *_= (2^*P *^- 1) * *r *.

Table 7 shows the number of tree searches necessary for different numbers of partitions and taxa. The number of bootstrap replicates was kept constant with 100. PBS generally needs the fewest tree searches and thus the shortest computation time to provide the raw data. PABA generally needs the most time except when the number of bootstrap replicates is smaller than the number of PBS tree searches. In this case NDI/PABSA needs more tree searches. However, a complete NDI/PABSA approach also comprises the tree searches needed for PBS and LILD approaches. If the significance test presented herein is used or different strategies to analyze the data, the number of tree searches increases accordingly. For example, for each data set herein (1 untreated, 3 treated and 99 resampled) a minimum of 15,765 tree searches is necessary. However, due to programming the PAUP files and downstream calculations of the values it was much more efficient to conduct for each node a constrained and anti-constrained search individually, although this meant to search for the best tree 50 times. Thus, 16,500 tree searches were performed for each data set and the total number of tree searches conducted was 1,699,500.

**Table 7:** Number of tree searches for each approach needed given different numbers of taxa and partitions.

***# Taxa***	**PBS**	**LILD **	**NDI**	**NDI/PABSA**	**PABA**
*4 partitions*					
*25*	23	92	115	345	1,500
*100*	98	392	490	1,470	1,500
*250*	248	992	1,240	3,720	1,500
*25 partitions*					

*25*	23	575	598	7.6*10^11^	3.3*10^12^
*100*	98	2,450	2,548	3.2*10^12^	3.3*10^12^
*250*	248	6,200	6,448	8.2*10^12^	3.3*10^12^
*100 partitions*					

*25*	23	2,300	2,323	2.7*10^33^	1.2*10^34^
*100*	98	9,800	9,898	1.2*10^34^	1.2*10^34^
*250*	248	24,800	25,048	3.0*10^34^	1.2*10^34^

For BS based calculations it is assumed that only the nodes of the best tree are analyzed and no additional ones. For PABA the number of bootstrap replicates was kept constant with 100. NDI = only NDI is determined; NDI/PABSA = NDI and all lower orders of additions are also determined, a full-blown PABSA approach.

Herein all data were obtained using the parsimony criterion. However, all these approaches can also be used using the likelihood criterion, because they employ either differences in tree lengths or bootstrap values, which can be obtained using Maximum Likelihood. However, no maximum likelihood program exists to date, which employs likelihood substitution models for morphological data. Such models were developed for Bayesian inferences [94]. PABA was actually developed using maximum likelihood bootstrap values [5]. PBS likelihood derivates have also been proposed and used [53,74]. NDI/PABSA and LILD values could be obtained in a similar way using Maximum Likelihood. However, given the sheer amount of tree searches with more than 1.5 millions conducted in this study maximum likelihood analyses would have been too computational intensive to be performed in reasonable time despite the small number of 21 operational taxonomic units (OTUs).

All these methods can also be inferred using Bayesian inferences and in the case of PABA posterior probabilities instead of bootstrap values. However, for the BS based approaches it has first to be tested whether the harmonic mean tree length or the best tree length of the equilibrium has to be used. The first one is, for example, used for Bayes factor tests [e.g., [95]]. As for Maximum Likelihood the time to conduct the analyses presented herein would not have been reasonable. The total number of Bayesian inferences to be conducted would have been at least 78,795. This number is much smaller than that for the parsimony criterion, because the posterior probability distribution and thus the bipartition table is already determined during the unconstrained tree search [e.g., [96]]. However, assuming on average the short time of only 10 minutes for each Bayesian inference the time to complete the tree searches would have taken more than 500 days.

### Conclusion

Paedomorphosis can influence the phylogenetic reconstruction of salamander phylogeny even in combination with a large molecular data set. The impact is less severe than in the morphological alone analysis, but still three of the four paedomorphic taxa included in this study are affected. To ameliorate the negative impact of paedomorphosis the strategy to exclude paedomorphic character traits is preferable over the strategy to code the adult morphology of paedomorphic taxa as unknown because it deteriorates the phylogenetic signal in the morphological partition less severely. Nonetheless, both strategies reduce the conflict at nodes directly affected by paedomorphosis. Concerning the molecular data, RAG1 seems the gene with the highest potential to unravel salamander phylogeny. The rRNA partition is too conserved and the mtDNA data too variable. The different approaches to detect conflict are generally correlated in their results. NDI/PABSA and PABA are more conservative than PBS and LILD. Therefore, these approaches are preferable and with respect to computation time NDI outperforms PABA.

## Methods

### The data set and its partitions

The data sets of Wiens et al. [2] and Weisrock et al. [37] were compiled herein to achieve a larger data set in terms of character positions. Both data sets employed the nuclear rRNA data set published by Larson and Dimmick [35], which was also included in this study. The rRNA partition comprises sequence information of the small and the large subunit of the nuclear rRNAs [35]. In contrast to the analyses of Weisrock et al. [37], which used the morphological data set published by Larson and Dimmick [35] comprising only 32 characters, Wiens et al. [2] established a new morphological data set of 326 characters. Therefore, I used this more recent and comprehensive data set of Wiens et al. [2] in the analyses present herein. Finally, the RAG1 data set of Wiens et al. [2] and the mtDNA of Weisrock et al. [37] were also incorporated. The mtDNA partition included COI (subunit one of cytochrome *c *oxidase), ND1, ND2 (subunits one and two of NADH dehydrogenase), tRNA^Ala^, tRNA^Asn^, tRNA^Cys^, tRNA^Gln^, tRNA^Ile^, tRNA^Met^, tRNA^Trp^, tRNA^Tyr ^and the origin for light-strand replication [37]. In the compilation of the data set only OTUs were used, which covered all four partitions (morphology, RAG1, rRNA and mtDNA). However, to achieve a more representative coverage of salamanders I used composite OTUs like Wiens et al. [2]. In the Figs. 1, 3, 4, 5 the composite OTUs are indicated by either a representative species or genus name except for the outgroup OTUs, which are indicated by a higher taxonomic level. The composite OTUs are as follows: Anura (morphology: *Discoglossus jennae*; RAG1, rRNA, mtDNA: *Xenopus laevis*), Gymnophiona (morphology, RAG1: *Dermophis mexicanus*; rRNA, mtDNA: *Typhlonectes compressicaudata*), *Ambystoma gracile *(rRNA, mtDNA: *A. tigrinum*), *Ambystoma opacum *(rRNA, mtDNA: *A. californiense*),*Amphiuma pholeter *(rRNA, mtDNA: *A. tridactylum*), *Desmognathus ochrophaeus *(RAG1: *D. quadramaculatus*), *Dicamptodon aterrismus *(morphology: *D. ensatus*; RAG1: *D. tenebrosus*),*Hynobius nebulosus *(mtDNA: *H. leechi*), *Necturus beyeri *(morphology: *N. maculosus*),*Pseudoeurycea rex *(morphology: *P. werleri*), *Pseudotriton montanus *(rRNA: *P. ruber*), *Rhyacotriton variegatus *(morphology: *R. olympicus*; rRNA: *R. kezeri*), *Taricha rivularis *(morphology: *T. torosa*). All other OTUs were not composite ones. For accession numbers of sequence data and data sets refer to Wiens et al. [2] and Weisrock et al. [37].

Data sets can be partitioned in numerous ways, especially in the case of molecular data sets. For example, molecular data sets can be partitioned based on genes, codon positions, secondary structure features or certain properties such as substitution rates [e.g., [17,51]] and in the most extreme down to single character positions. However, the smaller a partition gets the larger becomes the random error. Furthermore, for some of the procedures invoked in this study too many partitions are not computable in reasonable time. Herein I was interested in the conflict between morphological and molecular data due to the process of paedomorphosis. Thus, the morphological data were assigned to one partition. However, the molecular analyses of Wiens et al. [2] and Weisrock et al. [37] were quite different in their results indicating conflicts also between them. Both data sets had the rRNA data in common, but used either RAG1 or mtDNA. Therefore, the molecular data was split into three partitions accordingly based on the 'gene' assignment.

### Phylogenetic Analyses

Phylogenetic analyses of the complete data set as well as of all possible combinations of partitions were conducted using the parsimony criterion in PAUP4b11 [97]. In this data set these are: morphology, RAG1, rRNA, mtDNA, morphology + RAG1, morphology + rRNA, morphology + mtDNA, RAG1 + rRNA, RAG1 + mtDNA, rRNA + mtDNA, morphology + RAG1 + rRNA, morphology + RAG1 + mtDNA, morphology + rRNA + mtDNA and RAG1 + rRNA + RAG1.

The weighting strategies for morphological characters employed by Wiens et al. [2] were adopted here to facilitate comparability. 20 multistate characters expressing quantitative variation along a single axis (e.g., length of a structure) were treated as ordered. 20 binary characters showing variation within a species were weighted using the frequency step-matrix approach [98,99] and 1 polymorphic multistate character was coded using the polymorphic method [98,99]. The remaining 285 morphological and all molecular characters were unordered and equally weighted. To balance the maximal step cost of 100 in the step matrices, the 21 characters employing step matrices got a character weight of 1 and all others one of 100 [for detailed discussion see [2,98,99]]. Heuristic searches with 10 replicates of random taxon addition and TBR branch swapping were performed to reconstruct the most parsimonious trees. Congruence between partitions was also tested using the ILD test [41] with 1,000 replicates. Each partition was compared with each possible combination of other partitions.

### BS based approaches

For the 50 different nodes obtained in the most parsimonious trees of the different combinations of partitions (Figs. 1, 3, 4, 5) BS values were calculated obtaining most parsimonious trees based on the procedures described in the subsection "Phylogenetic analyses" and constrained or anti-constrained tree searches for all possible combinations of partitions [67,68]. For PBS values, each individual partition was optimized on the constrained and anti-constrained most parsimonious trees obtained for the complete data set for each node. The significance of each BS and PBS value was tested using the Templeton [70] test [74]. Furthermore, NDI, PABSA_3_, PABSA_2 _and LILD were calculated for each node using equations 2–3 [40,63].

### PABA

To assess congruence between partitions node-by-node Struck et al. [5] proposed an approach based on the alteration of BP values on a given node as partitions are added. Therefore, the BP bipartition table of each partition and possible combination of partitions has to be determined. The same settings as for the phylogenetic analyses were used in the bootstrapping analyses with 1,000 replicates based on the maximum parsimony criterion. The general PABA approach is outlined below. For a detailed example see Struck et al. [5].

1) Determine BP bipartition table for each partition and all possible combinations of partitions. Because the bipartition tables in PAUP were restricted to show only BP values of 5 or higher all BP values ≤ 5 were set to 5 (thus the maximum of *δ *is 95).

2) For each node of interest, calculate the alteration, *δ*, of BP values when a partition is added to an existing data set for all possible combinations and orders of partition addition.

3) Calculate for each given node and partition the mean *δ *at each possible position of addition. *δ *is not included in calculating the mean if and only if both the before and after BP value is either 100 (*δ *= 0) or ≤ 5 (*δ *= 0). In the case of an already maximally supported node (BP = 100), further increase of BP value cannot be achieved, although the underlying phylogenetic signal may still change. Similarly, in the case of a minimally supported node (BP ≤ 5), further decrease can also not be achieved.

Alterations in BP support were examined for the 50 nodes of interest using the Java application PABA Explorer b0.1 (Meyer & Struck, pers. comm.; available from the author on request) and Microsoft Excel.

For both PABA and the BS based approaches, the average contribution of a partition to any set of nodes can be estimated by calculating the mean value of all *δ*, NDI, PABSA_3_, PABSA_2_, LILD or PBS values belonging to this set of nodes as well as to the partition. For PABA, this is in contrast to the average calculation of Struck et al. [5], which was based on the mean *δ *values of the nodes and not the actual *δ *values. Thus, the former approach was a weighted averaging approach, whereas this one is unweighted.

### Strategies ameliorating the effects of paedomorphosis

Though I acknowledge that individuals and taxa are always patchworks of paedomorphic and non-paedomorphic character traits and can never be themselves paedomorphic, herein the term paedomorphic taxon refers to taxa possessing a high degree of paedomorphic character traits. Wiens et al. [2] proposed two different strategies to ameliorate the negative impact of paedomorphosis. One was to code the adult morphology of paedomorphic taxa as unknown, because the sexual mature stages of metamorphosing and paedomorphic species are not comparable ontogenetic stages (S1). The other one was to exclude paedomorphic character traits from the data sets, if they can be identified (S2). Thus, in this study as in the one of Wiens et al. [2] both the characters 1–317 coding for adult morphology were treated as unknown in the supposedly paedomorphic taxa *Amphiuma means*, *Amphiuma pholeter*, *Necturus beyeri*, *Siren intermedia*, *Cryptobranchus alleganiensis*, and *Andrias davidianus *(S1) and 30 supposedly paedomorphic character traits were excluded (S2). Wiens et al. [2] identified paedomorphic character traits based on the presence of states in both larvae of metamorphosing taxa and adults of paedomorphic taxa prior to any phylogenetic reconstruction. To compare these different strategies as well as the combination of the two strategies (S12) based on a statistical rigor the Wilcoxon signed rank test was used. Therefore, differences between the data set treated by the particular strategy and the untreated data set were determined at each node for both the PABA and the BS based values. Positive values indicate a decrease in conflict using the particular strategy and negative ones an increase. These values were ranked according to their absolute value, i.e. without their algebraic signs. The sum of the ranks of the positive values as well as the sum of the negative ones was calculated. The Wilcoxon signed rank test can exhibit if significantly more positive or negative changes occurred based on their rank sum using a *z *test. The values for the treated data sets were obtained the same way as they were for the untreated one.

### Random partitioning of the data set

To test the significance of the PABA and the BS based values 99 data sets were generated encompassing partitions of the same size as the morphological, RAG1, rRNA and mtDNA partition, but the positions of the data set were randomly assigned to these partitions. This means, the complete data set remains the same though the positions within the partitions have changed. The exact procedure was as follows:

1.) Generate 99 data sets with positions randomly assigned to the partitions.

2.) Repeat for each resampled data set all BS based approaches and PABA to calculate all values anew.

3.) For each individual test value of PABA, NDI/PABSA, PBS or LILD generate the distribution based on the 99 resampled values.

4.) Determine the two-sided test probability *p *of the particular value.

If *p *is smaller or equal to 0.05 then the particular value is significantly different from a randomly assembled partition of the same size. This means, the value obtained cannot be achieved by chance and thus is due to the particular partition. For PABA the distribution has been centered at zero. Examples how to conduct all these analyses are provided in the Additional files 1, 2, 3, 4, 5, 6, 7, 8.

## Competing interests

The author(s) declare that they have no competing interests.

## Supplementary Material

Additional file 1 Detailed description of the analyses with line codes for PAUP. The files contains a detailed description of the generation of pseudosamples, the conducted PAUP analyses with line codes, the PABA and BS based calculations and the conduction of a WRST test. Click here for file

Additional file 2 Excel spread sheet, how to generate random partitions. This file gives an example how random partitions can be generated using Microsoft Excel. Click here for file

Additional file 3 Example log file of PAUP. This file contains the log output of PAUP, which is used for the PABA analyses using PABA Explorer and Microsoft Excel. Click here for file

Additional file 4 Excel spread sheet, how to calculate PABA results. This file gives an example how PABA results can be calculated using Microsoft Excel. Click here for file

Additional file 5 Tree lengths for the BS based approaches. This file contains the output of tree length for a specific node, partition and constraint generated by PAUP and used for the BS based calculations in Microsoft Excel. Click here for file

Additional file 6 Excel spread sheet, how to calculate BS based results. This file gives an example how BS based results can be calculated using Microsoft Excel. Click here for file

Additional file 7 Excel spread sheet, how to determine significance. This file gives an example how the significance of BS based and PABA results can be determined using Microsoft Excel. Click here for file

Additional file 8 Excel spread sheet, how to calculate WRST results. This file gives an example how the WRST results can be determined using Microsoft Excel. Click here for file
