# Supplementary material for: Data congruence, paedomorphosis and salamanders
Source: Front Zool. 2007 Oct 31;4:22. doi: 10.1186/1742-9994-4-22 (PMC2234405; doi:10.1186/1742-9994-4-22)
Supplement: Additional file 1 — Detailed description of the analyses with line codes for PAUP. The files contains a detailed description of the generation of pseudosamples, the conducted PAUP analyses with line codes, the PABA and BS based calculations and the conduction of a WRST test. [file 1742-9994-4-22-S1.pdf]

Detailed description of the analyses with line codes for PAUP

Generation of pseudosamples:

Random partitions of same sizes as the defined partitions morphology, RAG1, rRNA and mtDNA were generated using Microsoft Excel. As an example see Additional file 2. Therefore, 6,427 random numbers between 0 and 1 were generated in the first column using the command "*rand*". In the next column a continuous series of numbers from 1 to 6,427 was generated. 1 was assigned to the first cell of this series (e.g., B2) and then 1 was added in the next lower cell to the value of the next upper cell (e.g., *"=B2+1*" for C2, which equals 2, and then "*=C2+1*" for D2, which equals 3) and so on until all numbers till 6,427 were generated this way. Then only the values of both columns were copied in new columns and the columns were sorted based on the random numbers. This procedure results in a random distribution of the numbers from 1 to 6,427. The first 326 numbers of this random distribution were assigned to the 'morphological' partition, the next 1,530 numbers to the 'RAG1' partition, the next 2,742 numbers to the 'rRNA' partition and the last 1,829 numbers to the 'mtDNA' partition. Thus, random partitions of the same sizes than the defined partition were generated for 99 pseudosamples.

Conducting the PAUP analyses to obtain tree lengths and bootstrap support:

PAUP analyses were conducted for the original data set, the data sets treated by the different strategies ameliorating the impact of paedomorphosis and each pseudosample. After the data block with the original data of morphology, RAG1, rRNA and mtDNA (not shown here) it followed the assumptions block of Wiens et al. [1] regarding the morphological data. Paup line codes are always shown in italic and a smaller type set.

```
BEGIN ASSUMPTIONS;

        USERTYPE 'polymorph-1' STEPMATRIX = 5
                A     B     C     D     E
        [A]     .     25    50    75    100
        [B]     25    .     25    50    75
        [C]     50    25    .     25    50
        [D]     75    50    25    .     25
        [E]     100   75    50    25    .
        ;

        USERTYPE 'polymorph-2' STEPMATRIX = 5
                A     B     C     D     E
        [A]     .     25    50    80    100
        [B]     25    .     25    55    75
        [C]     50    25    .     30    50
        [D]     80    55    30    .     20
        [E]     100   75    50    20    .
        ;

        OPTIONS  DEFTYPE=unord PolyTcount=MINSTEPS ;

        TYPESET * UNTITLED  = unord: 1-9 11-12 14-24 26-30 33-52 54-57 59-63 65-68 70-108 110-116 118-130 132-
141 143-145 147-195 197-207 209-210 212 216 219 221-222 224-225 227-231 233-234 236 238-239 243-255 257 259-263
265-269 271-280 282-283 285-294 296-321 324-325 327-6427, ord: 58 117 131 142 146 196 208 211 220 223 232 237 256
258 264 270 284 295 323 326, 'polymorph-1': 10 13 25 31-32 53 64 69 109 213-215 217-218 226 235 240-242 322 ,
'polymorph-2': 281;

        WTSET * UNTITLED  = 100: 1-9 11-12 14-24 26-30 33-52 54-63 65-68 70-108 110-212 216 219-225 227-234
236-239 243-280 282-321 323-6427, 1: 10 13 25 31-32 53 64 69 109 213-215 217-218 226 235 240-242 281 322;

ENDBLOCK;
```

Then a Set block was included to define character sets either for the defined partitions of morphology, RAG1, rRNA and mtDNA or for randomly generated partitions of similar sizes. The Set block for the defined partitions:

Begin Set;

CharSet Mor= 1-326; [this defines the morphology partition]
CharSet RAG= 327-1856; [this defines the RAG1 partition]
CharSet 18S= 1857-4598; [this defines the rRNA partition]
CharSet MTD= 4599-6427; [this defines the mtDNA partition]
CharSet All= 1-6427; [this defines the complete data set]

End;

The Set block for the randomly generated partitions using Microsoft Excel (see above):

Begin Set;

[PS 1] [this is just an information, which pseudosample it is]
CharSet Mor= 220 979 ... 3010; [and 323 more position in this partition]
CharSet RAG= 2185 4677 ... 3680; [and 1,527 more position in this partition]
CharSet 18S= 5911 1344 ... 975; [and 2,739 more position in this partition]
CharSet MTD= 4479 1132 ... 1809; [and 1,826 more position in this partition]
CharSet All= 1-6427; [this defines the complete data set]

End;

Finally analyses were conducted to obtain tree lengths for the calculations of NDI/PABSA, PBS and LILD and bipartition tables of bootstrap support for PABA.

Begin Paup;

log file = Combined_Weighted_PS01.txt;
[PABA Explorer extracts from this file the bipartitions tables, compiles the tables of the different combinations of partitions into one table and converts them into a comma separated table, which can be imported into Microsoft Excel; in this example this would result in the PABA results of pseudosample 1.]

Outgroup 1;
Set Criterion=Parsimony;
Set Increase = Auto;
Set AutoClose = Yes;

[The constraints for the tree searches of the BS based approaches are defined; in this study a total of 50 constraints for 50 nodes recovered in the different combinations of partitions.]
Constraints Node01 (Monophyly) = [&U] (1-19 (20-21));
Constraints Node02 (Monophyly) = [&U] (1-18 (19-21));
...
Constraints Node50 (Monophyly) = [&U] (1-8 10-12 (9 13-21));

[!]
[!]
[! Combination: "MOR"]
[The exclamation mark is important, because it prompts PAUP to print the text in the square brackets into the log file; the phrase Combination: “MOR” tells PABA Explorer to assign the label MOR to the next bipartition table in the log file.]

Exclude All;
Include MOR;
[This ensures that only the positions of the 'morphological' partition are included and no others.]

Bootstrap Search=Heuristic NReps=1000/Enforce=No Converse=No AddSeq = Random NReps=10 Swap=TBR NChuck=100000 ChuckScore=1;
[I used chuckscores to prevent that the bootstrapping searches got stuck in large plateaus of equally parsimonious trees; I felt that 100,000 sampled trees in this case would be a large enough sample size at each replicate.]
ClearTrees;

[Now the tree lengths of the different constraint and anti-constraint trees for each of the 50 nodes are calculated for the BS based approaches.]
[!]
[!]
[! Constraints Node01]

HSearch /Enforce=Yes Constraints=Node01 Converse=No AddSeq = Random NReps=10 Swap=TBR Retain=No;
[This finds the best tree agreeing with the constraint Node01.]

PScores 1/ Total=No TL=Yes NonparamTest=No ScoreFile=Combined_Weighted_01_N_Mor.txt Append=Yes;
[The obtained tree length and only the tree length is written in this file and the tree lengths of each pseudosample is appended to this file. Thus, one file contained a list of all the tree lengths of the 99 pseudosamples for this node, constraint and partition. This file was then imported into Microsoft Excel for further calculations. Similar set ups were used for the defined partitions and the treated data sets.]

HSearch /Enforce=Yes Constraints=Node01 Converse=Yes AddSeq = Random NReps=10 Swap=TBR Retain=No;
[This finds the best tree disagreeing with the constraint.]

PScores 1/ Total=No TL=Yes NonparamTest=No ScoreFile=Combined_Weighted_01_A_Mor.txt Append=Yes;
[Similar file as above, but now for the anti-constraint of Node01 and 'morphology'.]

ClearTrees;

[This was repeated for each of the 50 nodes/constraints.]
[!]
[!]
[! Constraints Node 02]

HSearch /Enforce=Yes Constraints=Node02 Converse=No AddSeq = Random NReps=10 Swap=TBR Retain=No;

PScores 1/ Total=No TL=Yes NonparamTest=No ScoreFile=Combined_Weighted_02_N_Mor.txt Append=Yes;

HSearch /Enforce=Yes Constraints=Node02 Converse=Yes AddSeq = Random NReps=10 Swap=TBR Retain=No;

PScores 1/ Total=No TL=Yes NonparamTest=No ScoreFile=Combined_Weighted_02_A_Mor.txt Append=Yes;

ClearTrees;

…

```
[!]
[!]
[! Constraints Node 50]

HSearch /Enforce=Yes Constraints=Node50 Converse=No AddSeq = Random NReps=10 Swap=TBR Retain=No;

PScores 1/ Total=No TL=Yes NonparamTest=No ScoreFile=Combined_Weighted_50_N_Mor.txt Append=Yes;

HSearch /Enforce=Yes Constraints=Node50 Converse=Yes AddSeq = Random NReps=10 Swap=TBR Retain=No;

PScores 1/ Total=No TL=Yes NonparamTest=No ScoreFile=Combined_Weighted_50_A_Mor.txt Append=Yes;

ClearTrees;
```

[Then this setup was repeated for each possible combination of partitions except for the combination of all partitions.]

```
[!]
[!]
[! Combination: "RAG"]
Exclude All;
Include RAG;
Bootstrap Search=Heuristic NReps=1000/Enforce=No Converse=No AddSeq = Random NReps=10 Swap=TBR;
ClearTrees;
[!]
[!]
[! Constraints Node01]
HSearch /Enforce=Yes Constraints=Node01 Converse=No AddSeq = Random NReps=10 Swap=TBR Retain=No;
PScores 1/ Total=No TL=Yes NonparamTest=No ScoreFile=Combined_Weighted_01_N_RAG.txt Append=Yes;
HSearch /Enforce=Yes Constraints=Node01 Converse=Yes AddSeq = Random NReps=10 Swap=TBR Retain=No;
PScores 1/ Total=No TL=Yes NonparamTest=No ScoreFile=Combined_Weighted_01_A_RAG.txt Append=Yes;
ClearTrees;
[!]
[!]
[! Constraints Node 02]
HSearch /Enforce=Yes Constraints=Node02 Converse=No AddSeq = Random NReps=10 Swap=TBR Retain=No;
PScores 1/ Total=No TL=Yes NonparamTest=No ScoreFile=Combined_Weighted_02_N_RAG.txt Append=Yes;
HSearch /Enforce=Yes Constraints=Node02 Converse=Yes AddSeq = Random NReps=10 Swap=TBR Retain=No;
PScores 1/ Total=No TL=Yes NonparamTest=No ScoreFile=Combined_Weighted_02_A_RAG.txt Append=Yes;
ClearTrees;

...
[!]
[!]
[! Constraints Node 50]
HSearch /Enforce=Yes Constraints=Node50 Converse=No AddSeq = Random NReps=10 Swap=TBR Retain=No;
PScores 1/ Total=No TL=Yes NonparamTest=No ScoreFile=Combined_Weighted_50_N_RAG.txt Append=Yes;
HSearch /Enforce=Yes Constraints=Node50 Converse=Yes AddSeq = Random NReps=10 Swap=TBR Retain=No;
PScores 1/ Total=No TL=Yes NonparamTest=No ScoreFile=Combined_Weighted_50_A_RAG.txt Append=Yes;
ClearTrees;

[!]
[!]
[! Combination: "18S"]

...
...
...
[!]
[!]
[! Combination: "RAG18SMTD"]

...
```

[For the combination of all partitions the setup had to be altered, because for the calculations of PBS values the individual partitions have to be mapped on the trees obtained by the complete data set for each node/constraint.]

[!]
[!]
[! Combination: "MORRAG18SMTD"]
Exclude All;
Include MOR RAG 18S MTD;
Bootstrap Search=Heuristic NReps=1000/Enforce=No Converse=No AddSeq = Random NReps=10 Swap=TBR;
ClearTrees;

[!]
[!]
[! Constraints Node01]
HSearch /Enforce=Yes Constraints=Node01 Converse=No AddSeq = Random NReps=10 Swap=TBR Retain=No;
PScores 1/ Total=No TL=Yes NonparamTest=No ScoreFile=Combined_Weighted_01_N_MorRAG18SMTD.txt Append=Yes;

[In this part the 'morphological' partition will be mapped on the constraint tree obtained for the complete data set and Node01 and the tree length be saved.]

[!]
[!]
[! Partition Mor]

Exclude All;
Include Mor;

PScores All/ Total=No TL=Yes NonparamTest=No ScoreFile=Combined_Weighted_01_N_PBSMor.txt Append=Yes;

[The obtained tree length and only the tree length for the mapped 'morphological' partition is written in this file and the tree lengths of each pseudosample is appended to this file. Thus, one file contained a list of all the tree lengths of the 99 pseudosamples for this node, constraint and partition. This file was then imported into Microsoft Excel for further calculations. Similar set ups were used for the defined partitions and the treated data sets.]

[Now the 'RAG1' partition will be mapped.]

[!]
[!]
[! Partition RAG]
Exclude All;
Include RAG;
PScores All/ Total=No TL=Yes NonparamTest=No ScoreFile=Combined_Weighted_01_N_PBSRAG.txt Append=Yes;

[Now the 'rRNA' partition will be mapped.]

[!]
[!]
[! Partition 18S]
Exclude All;
Include 18S;
PScores All/ Total=No TL=Yes NonparamTest=No ScoreFile=Combined_Weighted_01_N_PBS18S.txt Append=Yes;

[Now the 'mtDNA' partition will be mapped.]

[!]
[!]
[! Partition MTD]
Exclude All;
Include MTD;
PScores All/ Total=No TL=Yes NonparamTest=No ScoreFile=Combined_Weighted_01_N_PBSMTD.txt Append=Yes;

Exclude All;
Include MOR RAG 18S MTD;
HSearch /Enforce=Yes Constraints=Node01 Converse=Yes AddSeq = Random NReps=10 Swap=TBR Retain=No;
PScores 1/ Total=No TL=Yes NonparamTest=No ScoreFile=Combined_Weighted_01_A_MorRAG18SMTD.txt Append=Yes;

[Now the same for the anti-constraint tree.]

```
[!]
[!]
[! Partition Mor]
Exclude All;
Include Mor;
PScores All/ Total=No TL=Yes NonparamTest=No ScoreFile=Combined_Weighted_01_A_PBSMor.txt
Append=Yes;

[!]
[!]
[! Partition RAG]
Exclude All;
Include RAG;
PScores All/ Total=No TL=Yes NonparamTest=No ScoreFile=Combined_Weighted_01_A_PBSRAG.txt
Append=Yes;

[!]
[!]
[! Partition 18S]
Exclude All;
Include 18S;
PScores All/ Total=No TL=Yes NonparamTest=No ScoreFile=Combined_Weighted_01_A_PBS18S.txt
Append=Yes;

[!]
[!]
[! Partition MTD]
Exclude All;
Include MTD;
PScores All/ Total=No TL=Yes NonparamTest=No ScoreFile=Combined_Weighted_01_A_PBSMTD.txt
Append=Yes;
```

```
Exclude All;
Include MOR RAG 18S MTD;
ClearTrees;
```

[This new set up is repeated for each of the 50 nodes/constraints.]

```
[!]
[!]
[! Constraints Node 02]
HSearch /Enforce=Yes Constraints=Node02 Converse=No AddSeq = Random NReps=10 Swap=TBR Retain=No;
PScores 1/ Total=No TL=Yes NonparamTest=No ScoreFile=Combined_Weighted_02_N_MorRAG18SMTD.txt
Append=Yes;

    [!]
    [!]
    [! Partition Mor]
    Exclude All;
    Include Mor;
    PScores All/ Total=No TL=Yes NonparamTest=No ScoreFile=Combined_Weighted_02_N_PBSMor.txt
    Append=Yes;
```

```
[!]
[!]
[! Partition RAG]
Exclude All;
Include RAG;
PScores All/ Total=No TL=Yes NonparamTest=No ScoreFile=Combined_Weighted_02_N_PBSRAG.txt
Append=Yes;

[!]
[!]
[! Partition 18S]
Exclude All;
Include 18S;
PScores All/ Total=No TL=Yes NonparamTest=No ScoreFile=Combined_Weighted_02_N_PBS18S.txt
Append=Yes;

[!]
[!]
[! Partition MTD]
Exclude All;
Include MTD;
PScores All/ Total=No TL=Yes NonparamTest=No ScoreFile=Combined_Weighted_02_N_PBSMTD.txt
Append=Yes;

Exclude All;
Include MOR RAG 18S MTD;
HSearch /Enforce=Yes Constraints=Node02 Converse=Yes AddSeq = Random NReps=10 Swap=TBR Retain=No;
PScores 1/ Total=No TL=Yes NonparamTest=No ScoreFile=Combined_Weighted_02_A_MorRAG18SMTD.txt
Append=Yes;

[!]
[!]
[! Partition Mor]
Exclude All;
Include Mor;
PScores All/ Total=No TL=Yes NonparamTest=No ScoreFile=Combined_Weighted_02_A_PBSMor.txt
Append=Yes;

[!]
[!]
[! Partition RAG]
Exclude All;
Include RAG;
PScores All/ Total=No TL=Yes NonparamTest=No ScoreFile=Combined_Weighted_02_A_PBSRAG.txt
Append=Yes;

[!]
[!]
[! Partition 18S]
Exclude All;
Include 18S;
PScores All/ Total=No TL=Yes NonparamTest=No ScoreFile=Combined_Weighted_02_A_PBS18S.txt
Append=Yes;

[!]
[!]
[! Partition MTD]
Exclude All;
Include MTD;
PScores All/ Total=No TL=Yes NonparamTest=No ScoreFile=Combined_Weighted_02_A_PBSMTD.txt
Append=Yes;

Exclude All;
Include MOR RAG 18S MTD;
ClearTrees;

…
```

```
[!]
[!]
[! Constraints Node 50]
HSearch /Enforce=Yes Constraints=Node50 Converse=No AddSeq = Random NReps=10 Swap=TBR Retain=No;
PScores 1/ Total=No TL=Yes NonparamTest=No ScoreFile=Combined_Weighted_50_N_MorRAG18SMTD.txt
Append=Yes;

        [!]
        [!]
        [! Partition Mor]
        Exclude All;
        Include Mor;
        PScores All/ Total=No TL=Yes NonparamTest=No ScoreFile=Combined_Weighted_50_N_PBSMor.txt
        Append=Yes;
        [!]
        [!]
        [! Partition RAG]
        Exclude All;
        Include RAG;
        PScores All/ Total=No TL=Yes NonparamTest=No ScoreFile=Combined_Weighted_50_N_PBSRAG.txt
        Append=Yes;
        [!]
        [!]
        [! Partition 18S]
        Exclude All;
        Include 18S;
        PScores All/ Total=No TL=Yes NonparamTest=No ScoreFile=Combined_Weighted_50_N_PBS18S.txt
        Append=Yes;
        [!]
        [!]
        [! Partition MTD]
        Exclude All;
        Include MTD;
        PScores All/ Total=No TL=Yes NonparamTest=No ScoreFile=Combined_Weighted_50_N_PBSMTD.txt
        Append=Yes;

Exclude All;
Include MOR RAG 18S MTD;
HSearch /Enforce=Yes Constraints=Node50 Converse=Yes AddSeq = Random NReps=10 Swap=TBR Retain=No;
PScores 1/ Total=No TL=Yes NonparamTest=No ScoreFile=Combined_Weighted_50_A_MorRAG18SMTD.txt
Append=Yes;

        [!]
        [!]
        [! Partition Mor]
        Exclude All;
        Include Mor;
        PScores All/ Total=No TL=Yes NonparamTest=No ScoreFile=Combined_Weighted_50_A_PBSMor.txt
        Append=Yes;
        [!]
        [!]
        [! Partition RAG]
        Exclude All;
        Include RAG;
        PScores All/ Total=No TL=Yes NonparamTest=No ScoreFile=Combined_Weighted_50_A_PBSRAG.txt
        Append=Yes;
        [!]
        [!]
        [! Partition 18S]
        Exclude All;
        Include 18S;
        PScores All/ Total=No TL=Yes NonparamTest=No ScoreFile=Combined_Weighted_50_A_PBS18S.txt
        Append=Yes;
```

```
        [!]
        [!]
        [! Partition MTD]
        Exclude All;
        Include MTD;
        PScores All/ Total=No TL=Yes NonparamTest=No ScoreFile=Combined_Weighted_50_A_PBSMTD.txt
        Append=Yes;

Exclude All;
Include MOR RAG 18S MTD;
ClearTrees;
Log Stop;
End;
```

<u>*Calculating the PABA results:*</u>

The log files obtained in these analyses were imported into PABA Explorer, a JAVA application available from the author on request. See Additional file 3 for an example of such a log file. PABA Explorer extracts the bipartition tables from this file and assigns the corresponding label of combination of partitions to the table. Then PABA Explorer compiles all tables into one table and translates the .*-bipartitions into a Hennig bracket format of bipartitions. For example, ..** would be translated into (1, 2)(3, 4). Furthermore, if the node is not present in the bipartition table of a combination of partitions 5 is put into the cell, because the lower limit of the bipartition tables in PAUP were set to 5. This table can be exported as a comma separated table, which can be imported into, e.g., Microsoft Excel. In Microsoft Excel the PABA calculations were conducted as described in the manuscript. In Additional file 4 an example spread sheet is given. In the calculations of the mean values of alteration two kinds of no alteration in support have to be differentiated. First, no alteration can occur as a partition is added, because the node remains maximally or minimally supported (i.e., 5 to 5 or 100 to 100). Second, the bootstrap support remains constant as a partition is added (e.g., 76 to 76). Only the latter 0 values will be included the calculation of the mean value to avoid a bias (see manuscript and [2]). To automatically differentiate between these two cases the following formula was used in Microsoft Excel:
=(Cell1-Cell2)
*[ABS(Cell2-IF(Cell1>Cell2,100,(IF(Cell1<Cell2,5,(IF(Cell2=5,5,100))))))
/ABS(Cell2-IF(Cell1>Cell2,100,(IF(Cell1<Cell2,5,(IF(Cell2=5,5,100))))))].
Cell1 is the value of data set after the addition of the partition and Cell2 before the addition. (Cell1-Cell2) calculates the actual alteration. This is multiplied with a division, which has the same formula for both the dividend and the divisor. This ensures that the division is 1 and does not alter the actual value of the alteration. However, due to the divisor this formula it is able to differentiate the 0 alterations. In the divisor different values are subtracted from Cell2 depending on its relation to Cell1 and the absolute value is taking. If Cell1 is larger than Cell2 100 is subtracted. If Cell1 is smaller than Cell2 5 is subtracted. In these two cases the divisor has a value, which is not 0, and the division is 1. If Cell2 is equal to 5 again 5 is subtracted from Cell2. However, in this case the divisor is 0 and thus the division is not possible. Excel states this in the cell by "*#DIV/0!*". In all other cases not covered yet, 100 is subtracted from Cell2. So if Cell2 is equal to Cell1, but not to100, the divisor is different from 0 and the division is 1. However, if Cell2 is equal to both Cell1 and 100 then the divisor is again equal to 0 and the division not possible ("*#DIV/0!*"). Thus, the differentiation is automatically possible.

Calculating the BS based results:
The files with tree length for each partition, node and constraint or anti-constraint were imported into Microsoft Excel. See Additional file 5 for such a file as an example. Then NDI/PABSA, LILD and PBS were calculated as described in the manuscript and exemplified in the spreadsheet of the untreated data set with the defined partitions morphology, RAG1, rRNA and mtDNA (Additional file 6). The analyses of the pseudosamples were conducted in a similar manner. However, the 99 pseudosamples were in the rows instead of the nodes and each node had its own spreadsheet. The actual calculations were the same as in the example.

Determining significance:
The results of the pseudosamples for an approach (i.e., PABA, NDI/PABSA, PBS or LILD) and a node (e.g., node01) were compiled into one spreadsheet (Additional file 7). It was counted how often the approach was applicable. For NDI/PABSA, PBS or LILD this was always 100, for PABA this could be less. The next step was to count how often the values equal to or larger than the original value were found in the 99 values of the pseudosamples plus the one original value if the original value was larger than the mean. If the original value was smaller than the mean it was counted how often values were equal to or smaller than the original one. Finally, this count was divided by the number of times the approach was applicable (e.g., 100) and then multiplied by 2 to implement a two-sided tail probability. Probabilities equal to or smaller than 0.05 were regarded as significant.

Determining WRST results:
Finally, I also want to provide an example how the WRST results were determined. The procedure was similar in all other cases. The example shows if the contribution of the morphological partition is beneficial or detrimental to the set of nodes of Fig. 1 based on the LILD approach (see Table 3 in the manuscript). First the LILD values were sorted based on their absolute value (see Additional file 8). Then the ranks were assigned with algebraic signs re-inserted. If two or more ranks had values with the same absolute value the average of these ranks were used in calculations of the rank sum for each value. For example, in Additional file 8 rank 9 has a value of 700 and rank 10 has value of -700. The absolute value of both is the same and thus for both the rank used in the calculations of either the positive or the negative rank sum, respectively, was 9.5. Based on sample size, expected mean value and standard deviation the *z*-value can be determined. Using the *z*-test calculator (http://changbioscience.com/stat/ztest.html) the two-sided tail probability was determined. For a more detailed discussion of WRST see Felsenstein [3] in the context of hypothesis testing.

References

1. Wiens JJ, Bonett RM, Chippindale PT: **Ontogeny Discombobulates Phylogeny: Paedomorphosis and Higher-Level Salamander Relationships**. *Syst Biol* 2005, **54**(1):91 - 110.
2. Struck TH, Purschke G, Halanych KM: **Phylogeny of Eunicida (Annelida) and Exploring Data Congruence using a Partition Addition Bootstrap Alteration (PABA) approach.** *Syst Biol* 2006, **55**:1-20.
3. Felsenstein J: **Inferring Phylogenies.** Sunderland, MA: Sinauer Associates, Inc.; 2003.
